# Modeling the cost-effectiveness of maternal acellular pertussis immunization (aP) in different socioeconomic settings: A dynamic transmission model of pertussis in three Brazilian states

**DOI:** 10.1016/j.vaccine.2020.09.008

**Published:** 2021-01-03

**Authors:** Paula M. Luz, Claudio J. Struchiner, Sun-Young Kim, Ruth Minamisava, Ana Lucia S. Andrade, Colin Sanderson, Louise B. Russell, Cristiana M. Toscano

**Affiliations:** a Instituto Nacional de Infectologia Evandro Chagas, Fundação Oswaldo Cruz, Rio de Janeiro, Brazil; b Escola de Matemática Aplicada, Fundação Getúlio Vargas, Praia de Botafogo, 190, Rio de Janeiro, Brazil; c Instituto de Patologia Tropical e Saúde Pública, Universidade Federal de Goiás, Goiania, Goiás, Brazil; d Seoul National University, Department of Healthcare Management and Policy, SNU Graduate School of Public Health, 1 Gwanak-ro, Gwanak-gu, Seoul 08826, South Korea; e Faculdade de Enfermagem, Universidade Federal de Goiás, Goiania, Goias, Brazil; f London School of Hygiene and Tropical Medicine, Department of Health Services Research and Policy, 15-17 Tavistock Place, London WC1H 9SH, United Kingdom; g University of Pennsylvania, Department of Medical Ethics and Health Policy, 423 Guardian Drive, Philadelphia PA 19104, USA

**Keywords:** Pertussis, Maternal immunization, Dynamic transmission models, Cost-effectiveness analysis

## Abstract

**Objectives:**

Using dynamic transmission models we evaluated the health and cost outcomes of adding acellular pertussis (aP) vaccination of pregnant women to infant vaccination in three Brazilian states that represent different socioeconomic conditions. The primary objective was to determine whether the same model structure could be used to represent pertussis disease dynamics in differing socioeconomic conditions.

**Methods:**

We tested three model structures (SIR, SIRS, SIRSIs) to represent population-level transmission in three socio-demographically distinct Brazilian states: São Paulo, Paraná and Bahia. Two strategies were evaluated: infant wP vaccination alone versus maternal aP immunization plus infant wP vaccination. Model projections for 2014–2029 include outpatient and inpatient pertussis cases, pertussis deaths, years of life lost, disability-adjusted life-years (DALYs) lost, and costs (in 2014 USD) of maternal aP vaccination, infant vaccination, and pertussis medical treatment. Incremental cost per DALY averted is presented from the perspective of the Brazilian National Health System.

**Results:**

Based on goodness-of-fit statistics, the SIRSIs model fit best, although it had only a modest improvement in statistical quantitative assessments relative to the SIRS model. For all three Brazilian states, maternal aP immunization led to higher costs but also saved infant lives and averted DALYs. The 2014 USD cost/DALY averted was $3068 in Sao Paulo, $2962 in Parana, and $2022 in Bahia. These results were robust in sensitivity analyses with the incremental cost-effectiveness ratios exceeding per capita gross regional product only when the probability that a pertussis case is reported was assumed higher than base case implying more overt cases and deaths and therefore more medical costs.

**Conclusions:**

The same model structure fit all three states best, supporting the idea that the disease behaves similarly across different socioeconomic conditions. We also found that immunization of pregnant women with aP is cost-effective in diverse Brazilian states.

## 1 Introduction

Currently, pertussis is endemic in all countries causing 16 million cases and ∼195,000 child deaths annually [1], with epidemic cycles occurring every 2–5 years [2]. Childhood immunization has led to longer intervals between pertussis outbreaks which indicates increased herd immunity [3], [4]. Also, a shift in the age distribution of pertussis towards older age groups (adolescents and young adults) has been reported in recent years in high-income countries [5]. Recently, however, many countries have reported a resurgence in pertussis cases and deaths, particularly among infants too young to receive their own vaccinations [6], [7].

Single-dose maternal acellular pertussis (aP) immunization during pregnancy, which confers immunity on the infant through transplacental antibody transfer and reduces infants’ exposure to infection by protecting their mothers, could efficiently prevent disease and death in infants. Countries’ existing maternal immunization programs, which already provide tetanus toxoid, offer a feasible platform to provide maternal aP immunization, although at additional cost. To date, economic analysis of maternal aP immunization have shown it to be a cost-effective strategy in high-income countries [8], [9], [10].

Brazil introduced pertussis wP vaccine into its routine childhood immunization program in 1973. Currently, the schedule includes a 3 dose-primary series of pentavalent vaccine including diphtheria, tetanus, whole-cell pertussis, *Haemophilus influenzae* type b, and Hepatitis B (DTwP-Hib-HB) recommended at 2, 4 and 6 months of age, followed by two booster doses with DTwP at 15 months and 4 years of age. In the National Health System, aP vaccines are available, free of charge, only for children at high risk of adverse reactions to the wP vaccine (preterm infants, children with severe neurological or cardiac conditions and those who have previously had severe adverse events following whole-cell pertussis immunization) [11]. Additionally, aP vaccines are available in private immunization clinics. In November 2014, for pregnant women, the Brazilian Ministry of Health replaced diphtheria-tetanus vaccine with a single dose vaccine of tetanus-diphtheria-acellular pertussis vaccine [12] purchased through the Pan American Health Organization Revolving Fund.

We tested three structures for dynamic transmission models to explore the cost-effectiveness of maternal immunization in different socioeconomic conditions. Because Brazil has state-specific demographic and surveillance data we tested these model structures against data for three Brazilian states that represent a range of socioeconomic conditions (Sao Paulo, Parana, and Bahia). These conditions are illustrative of upper to lower-middle income settings. Since many low- and middle-income countries (LMICs) do not have enough data, of sufficient quality, to allow the development of a country-specific dynamic transmission model, the aim of our analysis was to determine whether a single model structure could fit three diverse states, and thus might represent disease dynamics in other countries with similar socioeconomic conditions. Additionally, we used the models to project the health outcomes, costs and cost-effectiveness of introducing maternal aP immunization in these three states.

## 2 Methods

### 2.1 Model structures and parameters

We developed three population-level age-structured compartmental models of pertussis transmission dynamics. Each model is described by a system of differential equations with up to five compartments to describe disease natural history and four to describe childhood and maternal vaccination. Each of the compartments is subdivided into 13 age compartments (0–1 month, 2–3, 4–5, 6–8, 9–11, 12–23 months, 2–4, 5–9, 10–14, 15–19, 20–49, 50–79, 80+ years), which were parameterized with state population size, birth rate and age-specific death rates.

The epidemiologic models are extensions of the classic Susceptible-Infectious-Recovered (SIR) model that includes vaccine compartments describing routine infant vaccination in addition to, depending on model structure, two types of infection: primary (I) and secondary (Is). Susceptible individuals (S) of all ages can acquire infection from contact with an infectious person (I or Is). Time spent infected corresponds to the infectious period, during which transmission may occur according to age-specific force of infection. When infected individuals recover from primary infection (I), they initially have immunity against pertussis (R) but immunity may wane, depending on the model structure.

To identify the model structure that best fits the data for each state, three structures with different assumptions about immunity waning and repeat infections were tested. Our approach reflects uncertainty surrounding the epidemiology of pertussis [13], [14]. The SIR model assumes lifelong immunity and consequently no secondary infections occur. The SIRS model assumes that immunity wanes and that secondary infections are as infectious and symptomatic (and therefore reported to the same extent) as primary infections. The SIRSI model also assumes that immunity wanes but, compared to primary infections, secondary infections are as infectious but less symptomatic and therefore less likely to be reported. That is, the SIR model assumes lifelong immunity while the SIRS and the SIRSI models assume immunity wanes and that individuals become susceptible to repeat primary infections (model SIRS) or secondary infections (Is, model SIRSI).

Routine infant whole-cell pertussis (wP) vaccination occurs at 2–3, 4–5, 6–8, 9–11, 12–23 months depending on the observed probability of receiving each of the primary childhood doses. These probabilities determine the fraction of infants that moves from the susceptible (S) or the maternal immunization (Vm, see below) compartments to the vaccinated compartments (V1: first dose, V2: second dose, V3: third dose). Infants vaccinated against pertussis may be protected by vaccination (V1p) or not protected (V1n) depending on the primary vaccine failure probability (vf). From vaccine protected compartments (V1p, V2p, V3p), upon waning, they may become susceptible to infection (move to S). Immunity from both routine vaccination (V) and maternal immunization (Mv) wane at different rates.

Maternal immunization is modeled by assigning a fraction of newborns, based on vaccine coverage, into a maternally immune compartment (Vm) which is further subdivided into a protected (Vmp) and not protected (Vmn) depending on vaccine effectiveness. When maternal immunity wanes, those initially protected become susceptible to pertussis infection (S). All “not protected” compartments are as susceptible as S. All “protected” compartments are immune, with different waning immunity rates.

Force of infection was defined for each age group according to effective contact rates and number of infected people. We modeled the contact between age-compartments based on a contact matrix that describes the average number of contacts per day that each individual is likely to have with another individual. Since an empirically-derived matrix is not yet available for Brazil, we adapted one for a middle-income country, Poland [15], to the Brazilian scenario by adjusting the published matrix by the ratio of average household size (Poland::Brazil) and subsequently to each state (Brazil::State). The Polish matrix was chosen based on a qualitative assessment of the similarities of the contact patterns in the two countries. For use in the model, the original matrix, which used 5-year age groups, was condensed/expanded as necessary to map to the 13 age groups in the present study. Additionally, the matrix was corrected for reciprocity - that is, following prior studies, it was adjusted so that the off-diagonal elements were symmetric and the contact rate was the same for any two age-groups regardless of which group initiated contact [16].

The model includes parameters to quantify duration and degree of infectiousness; duration of natural, vaccine, and maternally acquired immunity; efficacy and coverage of infant and maternal vaccines; and the likelihood that whole-cell or acellular vaccine will fail to protect. Parameter values, ranges and sources are described in Table 1, a more thorough description is given in the Technical Appendix, Section #8.

**Table 1:** Model parameters: symbol, description, sources, and base case values (range).

Symbol	Description	Definitions and sources
Demography		
Births	Births	# of births/day, state data from the Brazilian National Institute of Geography and Statistics. See Technical Appendix, Section #1.
d	Ageing	1/duration of age group. For example, for the age group 10–14 years, duration is 5 years. Defined for age group i = 1–12, there is no ageing parameter for the last age group.
m	Mortality	Mortality rate from all causes, state-specific population data from the National Institute of Geography and Statistics and all-cause mortality data from the National Mortality System. See Technical Appendix, Section #1. Defined for age group i = 1–13.
Natural history		
fi	Force of infection	Time-dependent parameter based on age-specific contact rates and the pool of infectious individuals at any given time. Defined for age group i = 1–13.
c_i,j_	Contact rate between age group i (i = 1–13) and age group j (j = 1–13)	Polish POLYMOD matrix [15] adjusted for average state-specific household size. See Methods and Technical Appendix, Section #1.
pi	Transmission probability per contact	Initial values for each state based on [17] and then adjusted during model fitting, see Technical Appendix, Section #1. Defined for age group i = 1–13.
g	1/infectious period	1/25 days (21–28). Initial values based on [8] and then adjusted during model fitting. See Technical Appendix, Section #1 and #6.
wi	Waning of infection induced immunity	25 years (10–50). Derived during model fitting, see Technical Appendix, Section #1 and #6.
mpi	Pertussis-specific mortality	Mortality rate from pertussis, state-specific population data from the National Institute of Geography and Statistics and pertussis-specific mortality data from National Mortality System. See Technical Appendix, Section #2. Defined for age group i = 1–5.
r	Reporting probability for symptomatic cases	State-specific likelihood that an infection will be symptomatic/reported. Derived during model fitting, see Technical Appendix, Section #1 and #6. The reporting probability for secondary infections, applicable to model structure SIRSI, was assumed to be 1/10 of that of the primary infection.
Vaccine coverage		
vc1	Vaccine coverage wP, 1st dose	Probability of being vaccinated, estimated from the National Survey of Vaccine Coverage in State Capitals in Brazil in 2007. See Technical Appendix for details, Section #3.
vc2	Vaccine coverage wP, 2nd dose	Probability of being vaccinated, estimated from the National Survey of Vaccine Coverage in State Capitals in Brazil in 2007. See Technical Appendix for details, Section #3.
vc3	Vaccine coverage wP, 3rd dose	Probability of being vaccinated, estimated from the National Survey of Vaccine Coverage in State Capitals in Brazil in 2007. See Technical Appendix for details, Section #3.
vcm	Vaccine coverage aP, maternal	State-specific data from the Brazilian National Immunization Program, Section #3.
Vaccine effectiveness		
ve1	Effectiveness wP, 1st dose	0.68 (95%CI: 0.456–0.811) [18], [19]
ve2	Effectiveness wP, 2nd dose	0.92 (95% CI: 0.847–0.957) [18], [19]
ve3	Effectiveness wP, 3rd dose	0.99 (95% CI: 0.989–1.000) [18], [19]
vem	Effectiveness aP, maternal	0.91 (95% CI:0.82–0.95) [20]
Primary vaccine failure		
vfw	Proportion of vaccine failure, wP	0.1 (0–0.15) [21]
vfa	Proportion of vaccine failure, aP	0.1 (0–0.15) [16]
Immunity waning		
w1	Waning immunity of wP 1st dose	14.5 years (range 5–30 years). Derived during model fitting, see Technical Appendix, Section #1 and #6.
w2	Waning immunity of wP 2nd dose	14.5 years (range 5–30 years). Derived during model fitting, see Technical Appendix, Section #1 and #6.
w3	Waning immunity of wP 3rd dose	14.5 years (range 5–30 years). Derived during model fitting, see Technical Appendix, Section #1 and #6.
wm	Waning immunity of aP, infant*	3 months (range 2–4 months) [16]

* Waning of immunity in the infant; waning of immunity in the pregnant woman is not modeled. State-specific means that data or parameters are specific to each of the Brazilian states modelled in the present analysis: São Paulo, Paraná and Bahia.

### 2.2 Data

We modeled maternal aP immunization in three states: (a) São Paulo in Southeast Brazil, (b) Paraná in the South, and (c) Bahia in the Northeast. São Paulo is the most populous, the most developed, ranking 2nd in the country (out of 27) on the human development index and has the highest per capita income (Table 2) [22]. The gross regional product per capita (the equivalent of the gross domestic product for the regions of a country) mirrors the previous statistics and, in 2014, was 17,881 USD in São Paulo, 13,310 USD in Paraná and 6273 USD in Bahia [23]. Infant vaccine coverage was lowest in Bahia in the two nationally representative surveys (Table 2) [24], [25].

**Table 2:** Summary of the characteristics of the states.

	Sao Paulo	Parana	Bahia	Source
Population (millions, 2018)	45	11	15	[22]
Per capita income (monthly, Reais, 2017)	1712	1472	862	[22]
Employment (ind. ≥ 16 years of age, 2017)	72%	68%	40%	[22]
Human development index (HDI, 2010)	0.783	0.749	0.660	[22]
Relative HDI ranking (out of 27)	2nd	6th	22nd	[22]
Gross regional product per capita (USD)	17,881	13,310	6273	[23]
Vaccination				
1991 birth cohort, assessed 1996, by region	72.9#	87.1#	60.7#	[25]
2005 birth cohort, assessed 2007, by state capital	83.0*	97.7*	78.7*	[24]

# Percent of children surveyed who within 12–23 months of age had received all childhood immunizations included in the national immunization program.

* Percent of children surveyed who by 18 months of age had received all childhood immunizations included in the national immunization program.

### 2.3 Pertussis epidemiology

In Brazil, notification of pertussis is compulsory, and the clinical, epidemiologic and laboratory criteria used for case classification are standardized and periodically revised by the national public health surveillance system [11]. We undertook a comprehensive review of available demographic, epidemiologic, and mortality data for the three states (see the Technical Appendix, Section #2). Mandatory notification of pertussis cases has been in place since 1973 (in electronic format since 1998) and notified cases are available through the Brazilian National Notifiable Surveillance System (SINAN) [11]. Suspected cases are defined based on symptoms while confirmation of suspected cases can be based on clinical, laboratory, or epidemiological criteria [11]. We considered all confirmed cases, irrespective of confirmation criteria, and defined outpatient cases as all confirmed cases in the surveillance information system except for those indicated as requiring hospitalization. Hospitalized cases were obtained from the Brazilian Hospitalization Information System (SIH, Sistema de Informações Hospitalares do SUS) and were defined as all individuals hospitalized within SUS in which the main cause of hospitalization was reported as being pertussis. Since these represent only hospitalizations occurring within the public health system, we adjusted the number of hospitalizations by public health system (SUS) coverage in each state to estimate all hospitalizations. Deaths from pertussis were obtained from the Brazilian Mortality Information System (SIM, Sistema de Informação de Mortalidade).

Infant vaccination against pertussis was initiated in the 1970s with notable national increase in coverage after 1973 leading to a significant reduction in incidence during the 1990s. More recently, a significant increase in pertussis incidence has been observed in Brazil with the highest rates in infants younger than 6 months [4] and evidence of pertussis infection among adolescents and adults [26]. Evidence from nationwide studies show an increase in pertussis incidence as early as 2007 and peaking in 2010–2014 [4], [7], [27], [28], [29]. Some authors have suggested that it could be associated with decreased vaccination coverage in some parts of the country and with a switch from DTwPHib to DTwPHib-HBV in 2012 [4], [26], [28], [30]. A more recent study from São Paulo State, in the Southeast of Brazil, described similar findings with increase in cases in all age groups in 2011–2014 with a sharp decrease in 2015 [31]. As pointed out by others, the challenges to interpreting these trends include the recent introduction of new and more sensitive molecular diagnostic methods, including PCR in 2009, enhanced sensitivity of the surveillance system with selected hospital-based sentinel sites for severe pertussis surveillance, enhanced disease awareness as a result of the recent epidemic, and decrease in infant vaccination coverage over the years [4], [26], [28], [30], [31].

As mentioned in the Introduction, the diphtheria-tetanus vaccine was replaced with tetanus-diphtheria-acellular pertussis vaccine (Tdap) for pregnant women in 2014 [12]. The rationale for this strategy is to protect the infant too young to be vaccinated with transplacental antibody transfer from mother to infant, as well as to protect the mother and potentially reduce household transmission.

### 2.4 Costs

Details of the cost data are provided in the Technical Appendix (Section #4). Briefly, in the present, we included direct medical costs pertaining to pertussis disease and the costs of the vaccines. Costs of pertussis outpatient cases were estimated based on standardized guidelines for pertussis case management and included diagnostic exams, medical visits, and medications. Costs of inpatient pertussis cases, stratified by age sub-group and by patient outcome (survive/death) were derived from reimbursements paid for all pertussis hospitalizations in 2014 in the hospitals of the Brazilian National Public Health system (SUS), which covers 75% of the Brazilian population.

Brazil began maternal aP immunization in 2014 using a single dose vaccine of multivalent formulation (adolescent/adult TdaP) purchased through the Pan American Health Organization Revolving Fund. To the base price per dose (USD 8.9), freight and insurance charges (3%) and a service charge (4.25%) were added, bringing the total to USD 9.55. An additional 5% wastage rate, as recommended by WHO and varied between 0 and 15% in sensitivity analyses, was added (total cost USD 10). Delivery costs were not separately estimated for maternal aP immunization since Td is being replaced by TdaP, as such it is assumed that there is no incremental cost of delivering TdaP.

For infant wP vaccines, we used the 2014 listed dose price for the single dose pentavalent vaccine DTwPHib-HBV liquid formulation, which is purchased through the Pan American Health Organization Revolving Fund. To the base price per dose, freight and insurance charges (3%) and a service charge (4.25%) were added, totaling USD 2.71. An additional 5% wastage rate, as recommended by WHO and varied between 0 and 15% in sensitivity analyses, brings the cost for each vaccine dose to USD 2.84. The cost of delivering a dose of vaccine for any individual was obtained from a primary study conducted in 2013 in Brazil which estimated the cost of the immunization program in the country [32].

### 2.5 Model fitting

An important step towards documenting the credibility and usefulness of a model is the process of model fitting. This involves exploring the different model structures as well as different parameter values which are varied within a priori plausible bounds to identify combinations that provide a better fit between model predicted time series and observed data. Parameters whose values were highly uncertain, even after consulting the available evidence, were derived during model fitting; these included: reporting probability for symptomatic cases, a multiplier that scales the probability of infection per contact, waning of immunity from infection, and duration of infection.

As calibration targets, we initially used monthly age-specific observed data from official sources for each Brazilian state for the period 2000–2016 and subsequently aggregated the data into one annual time series of cases for each age group (<1, 1–9, and 10+ years of age) to ease computational burden. These calibration targets were fitted to model predicted age-specific time series of infections which were grouped into the corresponding age groups: <1 year, 1–9 years and 10+ years of age, for each Brazilian state (São Paulo, Paraná, Bahia). Further details on the calibration targets and model predictions as provided in the Technical Appendix, Section #6.

To assess goodness of fit, we used graphical displays and multiple goodness-of-fit (GOF) statistics including the mean absolute error (MAE), which measures the average magnitude of the errors in a set of predictions; the root mean square error (RMSE), which gives the standard deviation of the prediction error; the normalized root mean square error (NRMSE), given in percentage; and the ratio of the RMSE to the standard deviation of the observations (RSR). For all these measures, the lower the value, the better the fit.

### 2.6 Interventions, primary outcome and analysis

The strategies modeled were 1) routine childhood pertussis wP vaccination only and 2) routine childhood wP vaccination plus aP maternal immunization. The impact of each strategy was simulated over a time horizon of 16 years, from 2014 when maternal aP immunization was introduced in Brazil to the end of 2029. We assumed the perspective of the Brazilian National Health system that accounts for direct medical costs for vaccination and disease treatment. Health, cost and cost-effectiveness outcomes were discounted at 3%, and the costs were expressed in 2014 US dollars.

For the cost-effectiveness analysis, our primary outcome was cost per disability-adjusted life year (DALY) averted. The number of DALYs was calculated as the sum of the number of years of life lost due to premature death and the number of years lost due to disability in the case of outpatient/inpatient cases using published methods [33]. These calculations used the disability weights for pertussis obtained from the Global Burden of Disease study [34] and average Brazilian life-expectancy, 81.75 years, as recommended in recent Burden of Disease studies from Brazil [35]. Further details are given in the Technical Appendix, Section #5.

The incremental cost-effectiveness ratio (ICER) of maternal aP was calculated by dividing the difference in cost between the two strategies, in 2014 US dollars, by the difference in DALYs which yields the cost/DALY. The decision to adopt a new intervention can be evaluated by comparing cost/DALY to estimates of societal willingness-to-pay for additional health gains. For comparison with prior studies, we defined an intervention as cost-effective if its cost/DALY averted is less than the state’s per capita Gross Regional Product [23].

One-way sensitivity analysis was assessed for a range of parameters including vaccine effectiveness, waning of immunity from infection, childhood vaccines and maternal vaccine, likelihood that the vaccine will fail, duration of infection, reporting probability for symptomatic cases, disability weight, discount rate, and costs (outpatient, inpatient, vaccine administration costs, maternal vaccine cost). Probabilistic sensitivity analyses were not performed as per current recommendations for dynamic transmission models [36].

## 3 Results

### 3.1 Model structure

**Fig. 1:**
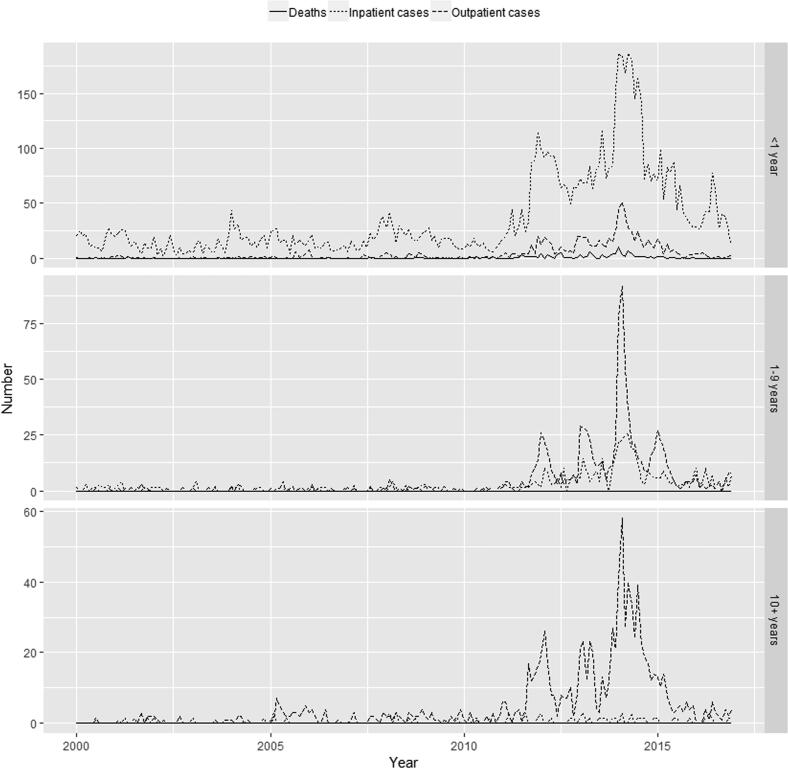
Age-specific (<1 year, 1–9 years and 10+ years of age) monthly pertussis deaths (solid), inpatient cases (dotted) and outpatient cases (dashed) from Brazilian official sources in São Paulo, 2000–2016.

**Fig. 2:**
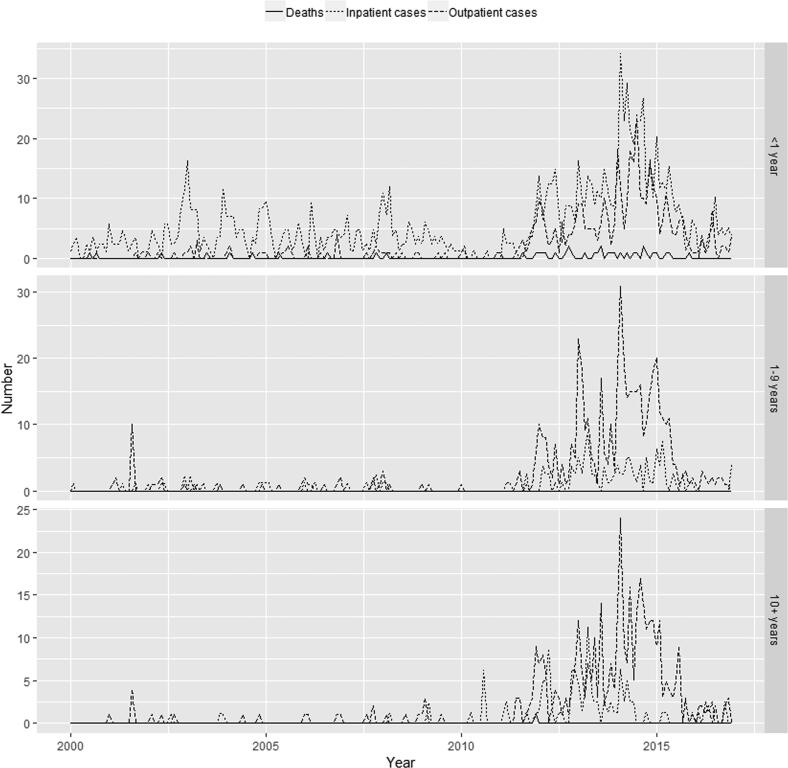
Age-specific (<1 year, 1–9 years and 10+ years of age) monthly pertussis deaths (solid), inpatient cases (dotted) and outpatient cases (dashed) from Brazilian official sources in Paraná, 2000–2016.

**Fig. 3:**
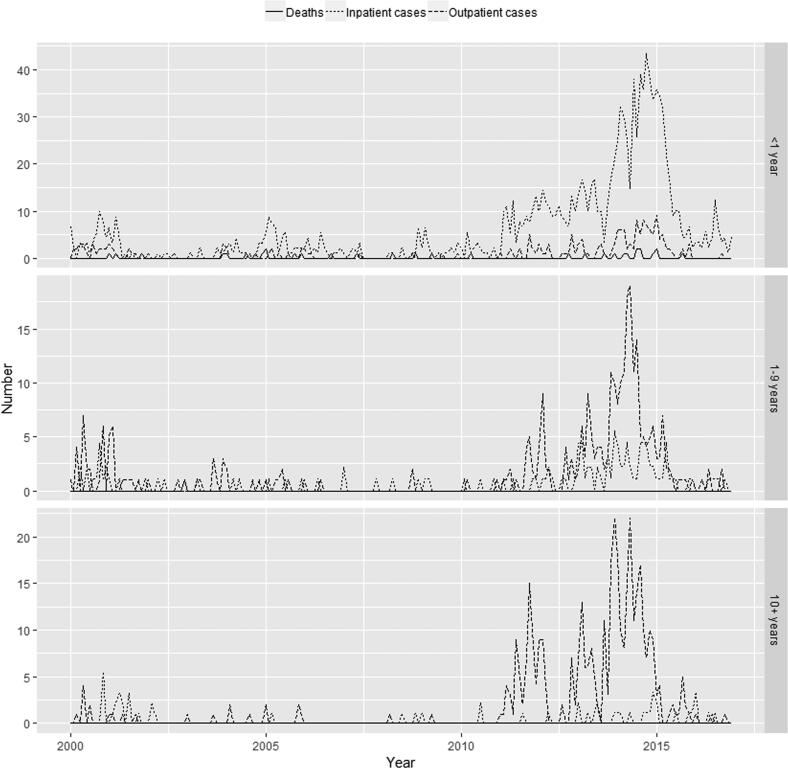
Age-specific (<1 year, 1–9 years and 10+ years of age) monthly pertussis deaths (solid), inpatient cases (dotted) and outpatient cases (dashed) from Brazilian official sources in Bahia, 2000–2016.

Fig. 1, Fig. 2, Fig. 3 show, by state, the monthly number of outpatient and inpatient cases and deaths by aggregated age groups (<1, 1–9, and 10+ years of age). Numbers of outpatient and inpatient cases and deaths were very low before 2010 and increased sharply between 2010 and 2014, as other epidemiologic studies from several regions in Brazil have shown [4], [29], [31]. Inpatient cases exceeded outpatient cases in those <1 year of age likely due to the higher probability of severe disease in this age group.

**Fig. 4:**
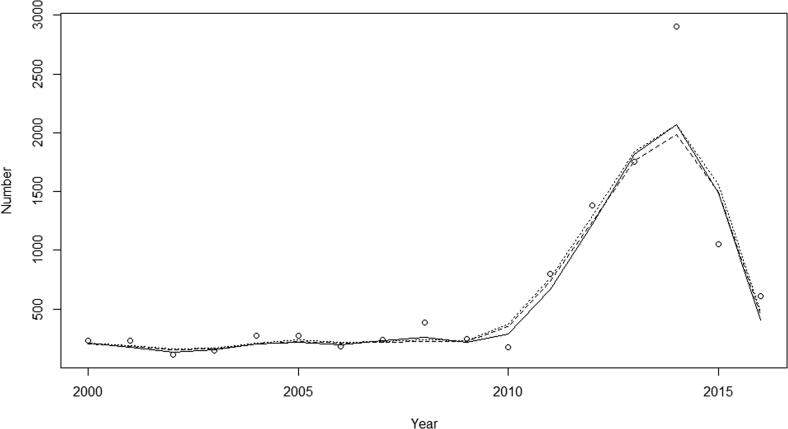
Comparison between the observed data (circles) and model projected outcomes by model structure SIR (solid), SIRS (dotted) and SIRSIs (dashed) in São Paulo, 2000–2016.

**Fig. 5:**
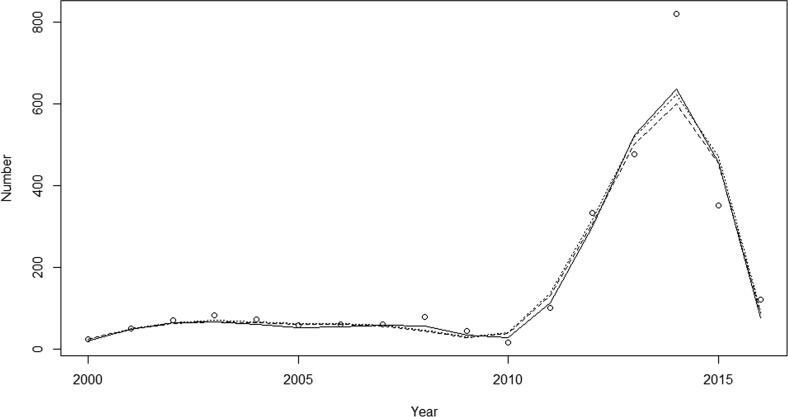
Comparison between the observed data (circles) and model projected outcomes by model structure SIR (solid), SIRS (dotted) and SIRSIs (dashed) in Paraná, 2000–2016.

**Fig. 6:**
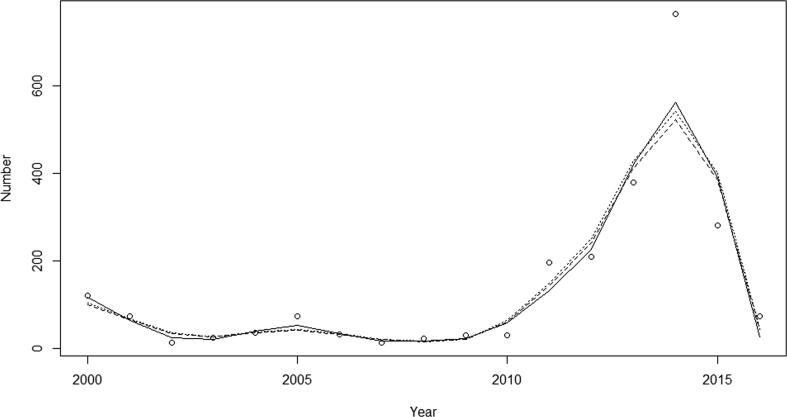
Comparison between the observed data (circles) and model projected outcomes by model structure SIR (solid), SIRS (dotted) and SIRSIs (dashed) in Bahia, 2000–2016.

Inspection of the graphical displays (Fig. 4, Fig. 5, Fig. 6) show that models fitted reasonably well to the observed data though all models miss the 2014 peak by a substantial amount (Fig. 4, Fig. 5, Fig. 6): the SIR produced the worst fit and the SIRS and the SIRSIs models fit best with small improvement in the fit statistics (Table 3). Among states, we see that the model for Paraná has the lowest NRMSE meaning that it had the best fit when compared to São Paulo and Bahia.

**Table 3:** Model goodness-of-fit statistics by model type and state: mean absolute error (MAE), root mean square error (RMSE), normalized root mean square error (NRMSE), and the ratio of the RMSE between the predictions and actual observation to the standard deviation of the observations (RSR).

	MAE	RMSE	NRMSE (%)	RSR
São Paulo				
SIR	141.7	239.5	65.4	0.65
SIRS	129.9	221.6	60.5	0.60
SIRSIs	121.5	221.5	57.8	0.58
Paraná				
SIR	20.5	35.9	45.3	0.45
SIRS	20.6	33.6	42.4	0.42
SIRSIs	19.8	32.3	40.7	0.41
Bahia				
SIR	25.2	47.9	61.5	0.62
SIRS	22.6	44.2	56.8	0.57
SIRSIs	21.0	42.3	54.3	0.54

Parameter values derived during model fitting included: reporting probability for symptomatic cases (São Paulo 3.4%, Paraná 2.5%, Bahia 1.4%), the multiplier that scales probability of infection (∼10×, not state-specific), waning of immunity from infection (∼25 years, not state-specific) and duration of infection (25 days, not state-specific).

### 3.2 Cost-effectiveness of aP immunization in the 3 states

The incremental cost-effectiveness of maternal aP immunization was USD 3068, 2962 and 2022 per DALY averted in São Paulo, Paraná and Bahia, respectively (Table 4). The following sections detail the results for each state and the impact of parameter uncertainty on the results.

**Table 4:** Impact of maternal aP on outpatient and inpatient cases, deaths, years of life lost, disability adjusted life-years and costs in São Paulo, Paraná, and Bahia, 2014–2029.

	São Paulo		Paraná		Bahia	
	No aP	With aP	No aP	With aP	No aP	With aP
Outpatient cases						
<1 year	15,237	3,547	3,756	1,877	2,902	1,242
1–9 years	46,957	25,066	9,702	7,877	7,014	5,540
10+ years	106,626	52,466	20,816	15,316	14,473	10,182
Total	168,820	81,078	34,273	25,069	24,389	16,966
Outpatient cases averted (N)		87,742		9,204		7,423
Outpatient cases averted (%)		52.0		26.9		30.4
Inpatient cases						
<1 year	22,856	5319	5629	2814	4359	1865
1–9 years	22,088	8810	5507	4043	3944	2707
10+ years	2452	1125	489	351	339	229
Total	47,399	15,250	11,626	7208	8643	4802
Inpatient cases averted (N)		32,148		4418		3841
Inpatient cases averted (%)		67.8		38.0		44
Deaths	301	70	138	66	201	85
Deaths averted (N)		232		72		116
Years of life lost (YLL)	24,288	5651	10,861	5,428	16,391	7011
YLL averted (N)		18,637		5433		9380
YLL averted (%)		76.7		50.0		57.2
DALYs						
<1 year	24,363	5668	10,881	5,439	16,405	7,017
1–9 years	138	66	25	25	25	13
10+ years	213	104	38	26	25	22
Total	24,712	5840	10,950	5493	16,453	7055
DALYs averted (N)		18,872		5458		9399
DALYs averted (%)		76.4		49.8		57.1
Costs (USD millions)						
Medical treatment	25.7	8.3	6.3	3.8	4.7	2.6
Vaccine	164.5	239.8	66.6	85.2	40.4	61.5
Total	190.3	248.2	72.9	89.0	45.1	64.1
Incremental costs		57.9		16.2		19.0
Cost (2014 USD) per DALY averted		3068		2962		2022

### 3.3 São Paulo

Maternal vaccination has an impact on all health outcomes, particularly deaths. With maternal vaccination, outpatient and inpatient cases decreased to 81,078 and 15,250 (52% of outpatient and 68% of inpatient cases were averted). Deaths in infants <1 year of age dropped to 70 and years of life lost due to pertussis dropped to 5651. Overall, 77% of the DALYs expected to be lost to pertussis were averted by maternal vaccination. Total costs were higher with maternal vaccination than for routine infant wP vaccination alone, with vaccine costs increasing by ∼USD 75 million while medical costs decreased to 8.3 million. In total, incremental costs of maternal vaccination amounted to USD 57.9 million.

One-way sensitivity analyses showed that, in order, reporting probability for symptomatic cases, infection duration, aP vaccine cost and infant wP vaccine coverage were the most influential parameters. None of these parameters, however, led to cost-effectiveness ratios that exceeded São Paulo’s gross regional product per capita (17,881 USD in 2014).

Reporting probability for symptomatic cases, a highly uncertain parameter, was varied within the range estimated in the model fitting process. A higher reporting probability (10.4%) implied a much higher burden of cases and deaths yielding significantly higher medical costs. In this scenario, aP becomes more cost-effective, at 392 USD/DALY averted. In contrast, when reporting probability was lower (1.2%), reported cases, deaths, and medical costs decreased substantially. In this scenario, vaccine costs represent a higher fraction of total costs and the cost of maternal immunization increases to USD 10,731 USD/DALY averted.

The impact of infection duration is similar in that when the infectious period is longer, more cases/deaths are expected, leading to a lower cost/DALY averted (when infection lasts 28 days, rather than 25 as in the base case, USD 2319/DALY). When the infectious period is shorter (21 days), there are fewer cases/deaths and lower medical costs, with the result that maternal immunization costs USD 9823/DALY. At a higher vaccine cost (15 USD) maternal immunization costs USD 4620/DALY (50% higher than base case) while at lower cost (5 USD) it costs USD 1773/DALY (half of base case).

Increasing infant wP vaccine coverage to levels reported in 1996 in São Paulo, which were higher than those in 2007 makes maternal immunization less attractive (USD 4719/DALY averted). Similarly, when infant vaccination is assumed more effective (for example, when wP vaccine failure is assumed to be zero), maternal immunization becomes less attractive (USD 4350/DALY averted).

### 3.4 Paraná

With maternal vaccination, 27% of outpatient and 38% of inpatient cases were averted. Deaths in infants <1 year of age dropped to 66 and years of life lost due to pertussis dropped by 50%. Overall, 50% of the DALYs expected to be lost to pertussis were averted by maternal vaccination. Total costs were higher due to vaccine costs. Paraná’s gross regional product in 2014 was 13,310 USD making maternal immunization in Paraná a cost-effective strategy, costing USD 2962 per DALY averted.

The same parameters that were most influential in São Paulo were also the most influential in Paraná. The reporting probability for symptomatic cases was the only parameter that led to significant changes with maternal immunization becoming more attractive when reporting probability was higher than base case (7.9%, USD 621/DALY averted) and not attractive when the reporting probability was lower than base case (0.4%, USD 18,604/DALY averted).

Increasing infant wP vaccine coverage to levels reported in 1996 in Paraná, which were higher than those in 2007 makes maternal immunization less attractive (USD 11,483/DALY averted). Compared to São Paulo and Bahia, the influence of infant wP vaccine coverage on the cost-effectiveness ratio was most striking for Paraná, a state where vaccine coverage has remained high over the years.

### 3.5 Bahia

Bahia was the state with the lowest estimated vaccination coverage and highest infant case-fatality. Nonetheless the results for the cost-effectiveness of maternal aP immunization were very similar to those in São Paulo and Paraná. In Bahia, the cost/DALY of maternal aP immunization was estimated at a third of Bahia’s gross regional product per capita (USD 6273 in 2014).

Again, the reporting probability for symptomatic cases was the most influential parameter, with maternal immunization costing USD 15,335/DALY averted when reporting probability was lower than base case (0.2%) and USD 395/DALY when reporting probability was higher than base case (5.2%). One-way sensitivity analyses again showed that, in order, infection duration, aP vaccine cost and wP vaccine coverage were also influential parameters though not to the point of making maternal vaccination not cost-effective.

## 4 Discussion

We developed and tested three dynamic transmission model structures (SIR, SIRS, SIRSI) parameterized to represent three distinct lower-middle and upper-middle income settings and used the best fitting model structure to project the cost-effectiveness of maternal aP immunization. We found that the same model structure fit all three settings, despite their different demographic and socio-economic conditions. The SIR model structure showed the poorest fit to the data suggesting that to adequately capture pertussis dynamics it is important to allow for waning immunity and repeat infections, as previous studies have suggested [14]. The difference in fit between the two models that incorporate waning (SIRS and SIRSIs) was minimal. That the same model structure provides the best fit to all three states best supports the idea that the disease behaves similarly across different socioeconomic conditions and that it may apply to other lower- and middle-income settings. Thus this analysis provides support for our use of an SIRSIs model fitted to Brazilian disease data, with appropriate changes in key parameter values, to evaluate the cost-effectiveness of maternal immunization [37].

In dynamic models, the rate at which susceptible individuals become infected depends on the number of infectious individuals in the population at any given time [38]. This force of infection is crucial to the evaluation of vaccine programs because vaccination not only reduces the incidence of disease in those immunized, by direct protection, but also indirectly protects nonvaccinated individuals against infection [38]. This indirect protection is often referred to as herd immunity [39], [40], [41]. In the simplest case, disease incidence should be reduced by a factor that is proportional to the product of vaccine coverage and vaccine efficacy [40]. However, a more realistic analysis, as Fine and colleagues suggests, should consider “(1) the distribution of vaccines and of disease risk in communities; (2) the nature of the immunity induced by the vaccine; as well as (3) the indirect protection of nonimmune persons by the presence of immune persons” [40]. Consideration of these factors implies that pertussis dynamics varies significantly over time and space.

Our models were developed following current recommendations for model conceptualization and validation. Recent literature has recommended that models aiming to assess the effect of vaccination should not apply the simulated vaccine program at birth nor at the end of the “vaccine schedule” (such as the end of the primary course) [14]. Accordingly, we have modeled vaccination by dose and age, incorporating both doses given at the recommended ages and those given later. This approach allowed for greater granularity in modeling vaccine coverage rates. Moreover, authors have suggested that models that fail to include fluctuations in vaccine coverage over time may not capture cohort effects, particularly when natural infection leads to long lasting immunity [14]. Our approach was to assume vaccine coverage changed over time as reported in nationally representative surveys of vaccine coverage in addition to published literature [24], [25], [42], [43], [44]. As for more generic characteristics, as suggested [14], [45], our models incorporate multiple age groups, assume age-specific contact rates, and use multiple sources of local data for parametrization and model fitting.

In agreement with other modeling and economic studies of pertussis resurgence and alternative vaccination strategies [14], [46], we found the reporting probability to be the most influential parameter. An analysis from the United States found that differences in the reporting probability (or reporting rate as it is usually denominated) implied a change in the decision whether or not to adopt maternal vaccination [8]. In a study from Australia, the reporting rate was the second most important parameter influencing the cost-effectiveness of maternal immunization [47]. When authors assumed that a higher fraction of cases was reported, maternal aP was more cost-effective. The reporting rate represents the fraction of infections that require medical attention and thus incur costs. There are many layers involved in the relationship between an infection and a reported case including probability of developing of symptoms, probability of seeking care, physician awareness and willingness to notify, testing likelihood and accuracy of the methodology, and notification infrastructure. As a result, one should use local data to estimate this parameter and/or explore its impact in sensitivity analysis as reporting rates from a particular study or population are not applicable to other settings [14].

For the three different settings here explored, we used model fitting processes to estimate the reporting probability for symptomatic cases that best fitted the observed data. Our estimates indicated that the reporting probability varied by state, lowest in Bahia and highest in São Paulo, which might reflect the fact that São Paulo was one of the first states to institute molecular diagnostic methods and, in general, has more referral centers and resources [31]. With the estimated reporting probabilities, maternal aP immunization was found to be a cost-effective strategy. A higher reporting probability than assumed in base case makes maternal aP immunization more cost-effective because the added costs of the vaccine are offset by the savings in treatment costs. In contrast, a lower reporting probability compared to base case leads to maternal aP immunization being less attractive. Epidemiologic studies of pertussis disease burden suggest that cases are significantly underreported even in high-income settings [48], [49], [50], [51] and thus that lower estimates for the reporting probability are more realistic.

We also found that higher infant wP vaccine coverage made maternal aP immunization a less attractive strategy. The reasoning for this finding is that as fewer notified cases occur, due to high infant vaccine coverage, there is less disease burden to be averted by maternal immunization. However, current infant wP vaccine coverage levels have not significantly decreased transmission, leading to an increased disease burden in recent years, in particular among infants <1 year who are most likely to be symptomatic, require hospitalization, and die [4], [31], [32]. Given currently observed pertussis incidence and infant coverage, maternal aP immunization would be a cost-effective strategy if the cost-effectiveness benchmark is each state’s GDP per capita, or even half of GDP per capita. Our results and conclusions are similar to those for Brazil as a whole [37]. Specifically, if infant wP vaccine coverage remains low, maternal aP immunization is cost-effective. At higher infant vaccine coverage rates (such as reported in 1996) the cost/DALY averted of maternal aP immunization is higher as there is less disease to be averted. In our analysis, the impact of higher infant vaccine coverage was most striking for Paraná, the state with highest coverage [24]. These findings evidence the contribution of herd immunity to the non-linear behavior of the dynamic model system [39].

Relatedly, in an accompanying paper [52], we compare maternal aP immunization cost-effectiveness results using a dynamic and a static model and highlight when the static model is likely sufficient for informing public health decisions. Results show that if infant vaccine coverage is low, then maternal immunization is cost-effective irrespective of model type. In contrast, when infant vaccine coverage is extremely high, then the dynamic transmission model is capable of showing how maternal immunization might not be cost-effective while the static model continues to suggest the strategy would be cost-effective. The rationale for implementing maternal aP immunization is direct protection of the newborns (0–2 months) through the maternal antibodies. As such, one could argue that having a maternal aP immunization in addition to infant vaccination could be valuable in settings where infant vaccine coverage cannot be expected to be maintained at a consistently high level over space and time. That is, if infant vaccine coverage is expected to vary, then maternal immunization acts as a backup, for safety, preventing infection among those who are most vulnerable to severe disease and death. For the present analysis, we reviewed available data on infant vaccine coverage for Brazil and its states and municipalities and found significant heterogeneity in space and time [24], [25], [43], [44], a scenario likely experienced in other low and middle income countries [53], [54].

We note a few important considerations on the concept of a “threshold proportion of immune persons that, if reached, could eliminate infection” (for a more complete assessment please refer to [40]). The herd immunity threshold is “an epidemiologic attribute with which to characterize particular infections” for which rough estimates have been generated [40]. In reality, however, heterogeneities in population demography, social structure, and contact rates (within and between age groups), the degree and duration of immunity elicited by pathogen and vaccine, as well as the stochastic nature of epidemic processes play a role in determining the level of vaccination required to eliminate disease in a particular time and place [40]. Though our levels of vaccine coverage were relatively high, for Paraná in particular, results show that it is likely far from decreasing incidence to the point of elimination. Moreover, studies have shown a significant reduction in vaccine coverage, to levels clearly below any threshold, at the municipal level (83% of Brazilian municipalities reporting DTP coverage of 95% in 2006 but only 55% in 2012) [29]. Incomplete vaccine coverage has been suggested as the reason for pertussis resurgence in Massachusetts as well [55].

Our study has several strengths and limitations that should be acknowledged. One strength was the use of local data to inform model fitting and parameter values. However, as observed in other settings [48], [49], [50], notified pertussis cases represent only a fraction of disease incidence which has influenced our fitting of the reporting probability parameter. In particular with regards to the 2014 increase in the number of reported cases, uncertainty about parameter values and model structure are among possible explanations for the under-fitting of the observed data. Of note is that, overall, all models under-fit the observed data for the age group <1 year meaning that maternal aP immunization cost-effectiveness ratios may well represent conservative estimates. Perhaps other modeling strategies, such as stochastic or individual-based models, could better address the weakness of the current work though these would add their own challenges such as high dimension of the parameter space, program coding complexity, increased computer processing time, and subsequent parameter non-identifiability. A limitation is that vaccine coverage rates used to inform the model were available for only a few time points and that heterogeneities in coverage [24], [44] were neglected. Moreover, for some parameters, such as the contact matrix, we had no local or Brazilian data and thus had to use data from countries with similar characteristics. The computational burden of fitting three different models to scarce observed data also limited the range of uncertainties that could be explored in sensitivity analyses. One example pertains to the reporting probability which was not age-specific and, for the SIRSI model that requires a reporting probability for secondary infections, an a priori assumption was made that its value be one-tenth of the reporting probability for primary infections. These assumptions, in turn, led to an age-dependent reporting probability effect in the SRISI model as it considers secondary infections that occur later in life. In addition, some aspects of pertussis natural history and the impact of vaccines, such as boosting or blunting of immunity from maternal antibodies, were not considered. Importantly, and following the recommendations highlighted in three reviews of economic analyses of pertussis vaccination strategies [46], [56], [57], limited understanding of important aspects of pertussis epidemiology suggests caution when extrapolating our results. More studies are needed to better inform the relationship between infection and case notification, the duration of immunity conferred by infection and vaccines, and how boosting of immunity may result from silent infections. Finally, though we did explore different model structures, we relied on an objective comparison of the goodness of fit of the three models, choosing the one with the best fit. The chosen model (SIRSIs) however, is less parsimonious than the SIRS model.

Nonetheless, dynamic transmission models are useful for projecting potential health outcomes and generating evidence for policy recommendations. We have shown that the SIRSIs model structure fit best the pertussis epidemiology of three settings that represent diverse socio-demographic conditions. It is thus reasonable to suggest that the same structure can represent disease transmission in a range of lower- and middle-income settings [37]. Our results also highlight the implications of the under-reporting of diseases which may lead to an underestimation of the value of reducing disease burden.

## Authors contributions

PML, CMT, SK, and LBR conceived the study, interpreted the results and wrote the paper. PML and CJS performed the analyses. SK, RM, ALA, CS, LBR and CMT collected the data and contributed to the analyses. CJS, SK, RM, ALA, and CS reviewed the manuscript.

## Funding

10.13039/100000865Bill & Melinda Gates Foundation Grant OPP1124529.

## Declaration of Competing Interest

The authors declare the following financial interests/personal relationships which may be considered as potential competing interests: Dr. Andrade has received lecture fees and travel grants from GlaxoSmithKline and Pfizer. Dr. Minamisava has received travel grants from GlaxoSmithKline. All other authors have no conflicts of interest.
