# Supplementary material for: Modeling the cost-effectiveness of maternal acellular pertussis immunization (aP) in different socioeconomic settings: A dynamic transmission model of pertussis in three Brazilian states
Source: Vaccine. 2021 Jan 3;39(1):125–36. doi: 10.1016/j.vaccine.2020.09.008 (PMC7738757; doi:10.1016/j.vaccine.2020.09.008)
Supplement: Supplementary data 1 [file mmc1.docx]

**Technical Appendix**

**A dynamic transmission model of pertussis in three Brazilian states: The cost-effectiveness of maternal acellular pertussis immunization (aP) in different socioeconomic settings**

Paula M. Luz, Claudio J. Struchiner, Sun-Young Kim, Ruth Minamisava, Ana Lucia S. Andrade, Colin Sanderson, Louise B. Russell, Cristiana M. Toscano

**Sections:**

**1. Model structure and parameters**

**2. Demographic and epidemiologic data**

**3. Vaccine parameters**

**4. Cost parameters**

**5. Model output**

**6. Model fitting procedures and results**

**7. Analysis**

**8. Symbols and notation**

**9. Model equations**

**10. References**

**1. Model structure and parameters**

***Model structure***

We developed an age-structured compartmental model of pertussis population-level transmission to project the clinical benefits, costs and cost-effectiveness of introducing maternal pertussis vaccination in three Brazilian states, each situated in a different region of the country: (a) São Paulo in the Southeast, (b) Paraná in the South, and (c) Bahia in the Northeast.

***Demographic model***

The demographic model includes state-specific population size, birth rate and age-specific death rates. The demographic model includes 13 age groups, as shown. Herein, age groups may also be referred to by their number.

| Age group # | Age group |  |
| --- | --- | --- |
| 01 | 0-1m |  |
| 02 | 2-3m |  |
| 03 | 4-5m |  |
| 04 | 6-8m |  |
| 05 | 9-11m |  |
| 06 | 12-23m |  |
| 07 | 2-4ys |  |
| 08 | 5-9ys |  |
| 09 | 10-14ys |  |
| 10 | 15-19ys |  |
| 11 | 20-49ys |  |
| 12 | 50-79ys |  |
| 13 | 80+ys |  |

***Epidemiologic model***

The epidemiologic models are extensions of the classic Susceptible-Infectious-Recovered (SIR) model that include vaccine compartments describing routine infant vaccination as recommend by the Brazilian National Immunization Program in addition to two types of infection: primary (Ip) and secondary (Is, only present for the SIRSI model, see details below). Susceptible individuals (S) of all ages can acquire infection from contact with an infectious person. Time spent infected corresponds to the infectious period, during which transmission may occur according to age-specific force of infection. When infected individuals recover from primary infection, they initially have immunity against pertussis (R) but immunity may wane depending on the model structure. The SIR model assumes immunity does not wane while the SIRS and the SIRSI models assume that immunity eventually wanes. As immunity wanes, individuals become susceptible again. In the SIRS model, as immunity wanes, individuals become susceptible to repeat primary infections while the SIRSI model assumes immunity wanes and individuals become susceptible to secondary infections (Is). Secondary infections are assumed to be as infectious but less symptomatic and therefore less likely to be reported. Time spent in each infected compartment (Ip and Is) corresponds to the infectious period, during which transmission may occur.

Routine infant vaccination occurs at 2-3, 4-5, 6-8, 9-11, 12-23 months based on the probability of receiving each of the primary childhood doses (coverage per dose by age) and vaccine efficacy when a fraction of the infants either from the susceptible (S) or the maternal immunization (Vm) compartments move to the subsequent vaccinated compartments (V1: first dose, V2: second dose, V3: third dose). Infants vaccinated against pertussis may be protected by vaccination (V1p) or not protected (V1n) depending on vaccine effectiveness. From vaccine protected compartments (V1p, V2p, V3p), upon waning, they become susceptible to infection. Vaccination is assumed to provide no additional protection to infected individuals beyond the immunity already conferred by the infection. Immunity from both routine vaccination (V) and maternal immunization (Mv) wane (or not, depending on model structure, see below), though at different rates, when individuals again become susceptible.

Maternal immunization is considered indirectly by assigning a fraction of the newborns, based on vaccine coverage, into a maternally immune compartment (Vm) which is further subdivided into a protected (Vmp) and not protected (Vmn) depending on vaccine effectiveness. When maternal immunity wanes, those initially protected become susceptible to pertussis primary infection (S). All “not protected” compartments are as susceptible as S, and thus are not subject to waning. All “protected” compartments are immune, waning immunity rates differ by compartment; when immunity wanes individuals move to S.

| Symbol | Description of model compartments |  |
| --- | --- | --- |
| Natural history |  |  |
| S | Susceptible to primary infection |  |
| I | Infected with primary infection |  |
| R | Recovered, immunity wanes |  |
| Ss | Susceptible to secondary infections (applicable to SIRSI model) |  |
| Is | Infected with secondary infections (applicable to SIRSI model) |  |
| Vaccine related |  |  |
| Vmp | Maternally vaccinated, protected |  |
| Vmn | Maternally vaccinated, not protected |  |
| V1p | Received V1, protected |  |
| V1n | Received V1, not protected |  |
| V2p | Received V2, protected |  |
| V2n | Received V2, not protected |  |
| V3p | Received V3, protected |  |
| V3n | Received V3, not protected |  |

**Alternative model structures**

Model schematic is shown in the Figure. The model is described by a system of differential equations with at least three compartments to describe disease natural history and four to describe childhood and maternal vaccination (see final section of Technical Appendix for model equations). The SIR model assumes that immunity from infection or vaccine is lifelong such that no secondary infections occur. The SIRS model assumes that immunity from infection/vaccine wanes over time such that individuals may again acquire an infection that is as symptomatic and therefore just as likely to be reported as primary infections. The SIRSI model again considers waning immunity from infection/vaccine but secondary infections, although just as infectious, are less symptomatic and therefore less likely to be reported.


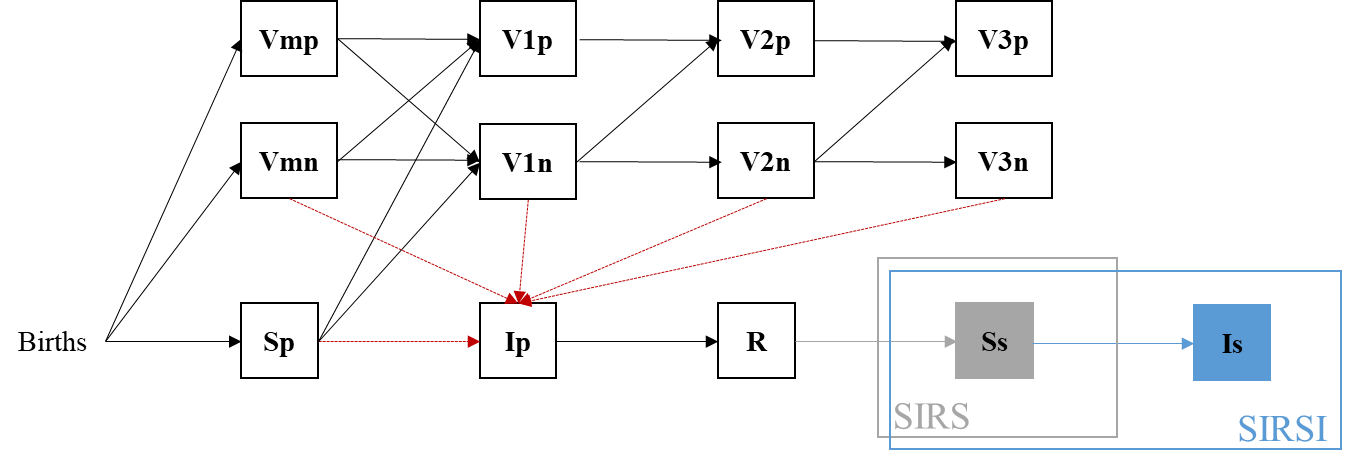


***Model parameters***

***Demographic model***

We modeled population growth, as experienced in each state, by incorporating the average annual birth cohorts (Bahia: 215000, Paraná: 160000, São Paulo: 560000, per year) and the age-specific mortality rates as derived from Brazilian sources, detailed in the next section.

**Table. Age- and state-specific monthly morality rates per 1000 population.**

| Age group # | Age group | Bahia | Paraná | São Paulo |
| --- | --- | --- | --- | --- |
| 01 | 0-1m | 9.41 | 8.51 | 6.44 |
| 02 | 2-3m | 1.66 | 2.25 | 1.19 |
| 03 | 4-5m | 1.13 | 1.50 | 0.69 |
| 04 | 6-8m | 0.71 | 0.57 | 0.33 |
| 05 | 9-11m | 0.57 | 0.26 | 0.30 |
| 06 | 12-23m | 0.19 | 0.21 | 0.12 |
| 07 | 2-4ys | 0.04 | 0.06 | 0.07 |
| 08 | 5-9ys | 0.03 | 0.03 | 0.02 |
| 09 | 10-14ys | 0.02 | 0.02 | 0.03 |
| 10 | 15-19ys | 0.05 | 0.08 | 0.12 |
| 11 | 20-49ys | 0.18 | 0.23 | 0.29 |
| 12 | 50-79ys | 1.22 | 2.31 | 2.07 |
| 13 | 80+ys | 5.28 | 10.71 | 9.74 |

***Epidemiologic model***

We used a mixture of clinical and epidemiologic literature values and model fitting to estimate the parameters. Natural history parameters included duration of the infectious state (g), waning of immunity acquired from infection (wi), pertussis-specific mortality (mp, derived from the state-specific epidemiologic information), and force of infection. Force of infection was defined for each age group according to effective contact rates and number of infected people. We modeled the contact between age-compartments based on a contact matrix that describes the average number of contacts per day per age group. Since an empirically-derived matrix is not yet available for Brazil, we adapted the one for Poland [1] to the Brazilian scenario by adjusting the published matrix by the ratio of the average household size of the two countries (Poland::Brazil) and subsequently to each state (Brazil::State). The Poland matrix was chosen based on a qualitative assessment of similarities of the countries. For use as model input, the initial 5-years age groups matrix were condensed/expanded so as to map to the 13 age groups chosen for the present study. Additionally, the matrix was corrected for reciprocity following procedures adopted in prior studies [2]. The following tables give the values for the state-specific contact rates.

There were no local estimates for the parameter transmission probability per contact by age group and we thus based our values on previous estimates [3]. However, we defined this as an uncertain parameter subject to adjustments in the model fitting process. Following, a table give the values for the transmission probabilities prior to model fitting.

There is evidence that immunity to pertussis from infection and vaccination wanes over time though the duration of immunity is highly uncertain. Prior modeling studies have assumed a range of values for these parameters, from lifelong immunity [4] to much shorter durations (<5 years). The exact rate at which these types of immunity wane are difficult to determine from epidemiological data. As such, our model’s estimates for waning immunity were chosen, within plausible ranges and restrictions, by hand to fit the data [5]. The values obtained for the duration of immunity were as follows: 25 years for infection and 14.5 years for each dose of infant Tdp vaccine.

Other vaccine-related parameters, in particular coverage and efficacy, will be described in the following sections. In sensitivity analyses, model parameters were varied within plausible ranges to determine the impact of the variation on the outcome of interest, the cost-effectiveness of maternal immunization.

Although pertussis is a notifiable disease in Brazil, similar to what has been shown in high-income settings [6-9], pertussis is underreported [10-12]. Accordingly, we assumed that a fraction of the model-generated infections would become symptomatic and therefore would become cases. In Section #6 we describe how the parameter, reporting probability for symptomatic cases, was estimated during model fitting. This reporting probability maps model predicted infections to symptomatic cases as indicated in the Figure.


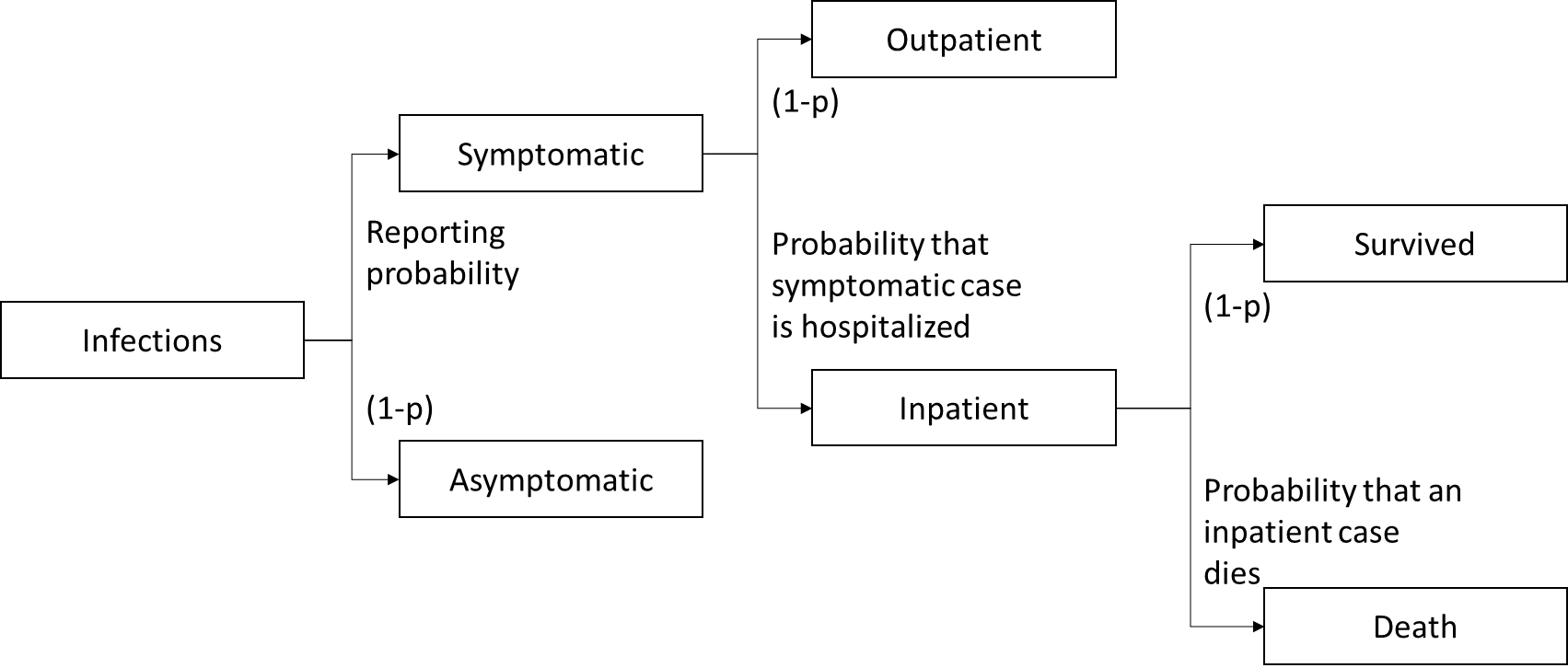


Symptoms were assumed to last for 2 weeks (4 weeks maximum). Symptomatic individuals might be hospitalized (inpatient) or not (outpatient) depending on their age (proportion of symptomatic that are hospitalized was assumed as 60% for <5 years, 0.8% for 5-19 years, and 3% for 20+ years [13]). Among infants <1 year of age, hospitalized individuals might recover (survive) or die. Case-fatality ratios were derived for each state from observed data by dividing, among those <1 year of age, the total number of deaths from pertussis by the number of hospitalized cases: Bahia 3.52%, Paraná 1.78%, São Paulo 1.07%. For all other ages, hospitalized cases were assumed to evolve to cure.

***Initial conditions***

Model’s initial conditions were derived from running the model without vaccination for 200 years, when endemic equilibrium was reached. Endemic equilibrium conditions were set for the year 1970 when the simulation started. wP infant vaccination was modeled as a function of time and assumed to have started in 1980. Vaccination coverage is then assumed to increase linearly over the years reaching the reported vaccination coverage as described in section 3 in year 1996. Between years 1996 and 2007, the years for which there is good quality data to inform model parameters, rates of vaccination are assumed to vary linearly between these two estimates. From 2007 onwards (until the end of the simulation in Dec 31 2029), vaccination coverage is assumed constant, at 2007’s level.

**Table. Daily contact rate by age group and state. These parameters are denoted by the symbol “c” which is followed by the group number of the age group that contacts a given age group. For example, the contact rate of age group 01 with age group 01 is denoted by c_01,01_.**

| Bahia |  |  |  |  |  |  |  |  |  |  |  |  |  |
| --- | --- | --- | --- | --- | --- | --- | --- | --- | --- | --- | --- | --- | --- |
| Age group # | **01** | **02** | **03** | **04** | **05** | **06** | **07** | **08** | **09** | **10** | **11** | **12** | **13** |
| 01 | 1.46 | 1.65 | 1.65 | 1.65 | 1.65 | 1.65 | 1.65 | 0.87 | 0.60 | 0.37 | 0.59 | 0.39 | 0.39 |
| 02 | 1.46 | 1.65 | 1.65 | 1.65 | 1.65 | 1.65 | 1.65 | 0.87 | 0.60 | 0.37 | 0.67 | 0.39 | 0.39 |
| 03 | 1.46 | 1.65 | 1.65 | 1.65 | 1.65 | 1.65 | 1.65 | 0.87 | 0.60 | 0.37 | 0.67 | 0.39 | 0.39 |
| 04 | 1.46 | 1.65 | 1.65 | 1.65 | 1.65 | 1.65 | 1.65 | 0.87 | 0.60 | 0.37 | 0.67 | 0.39 | 0.39 |
| 05 | 1.46 | 1.65 | 1.65 | 1.65 | 1.65 | 1.65 | 1.65 | 0.87 | 0.60 | 0.37 | 0.67 | 0.39 | 0.39 |
| 06 | 1.46 | 1.65 | 1.65 | 1.65 | 1.65 | 1.65 | 1.65 | 0.87 | 0.60 | 0.37 | 0.67 | 0.39 | 0.39 |
| 07 | 1.46 | 1.65 | 1.65 | 1.65 | 1.65 | 1.65 | 1.65 | 0.87 | 0.60 | 0.32 | 0.67 | 0.39 | 0.39 |
| 08 | 0.77 | 0.87 | 0.87 | 0.87 | 0.87 | 0.87 | 0.87 | 7.05 | 1.47 | 0.38 | 0.69 | 0.44 | 0.44 |
| 09 | 0.60 | 0.60 | 0.60 | 0.60 | 0.60 | 0.60 | 0.53 | 1.47 | 13.97 | 1.80 | 0.78 | 0.41 | 0.41 |
| 10 | 0.37 | 0.37 | 0.37 | 0.37 | 0.37 | 0.37 | 0.32 | 0.38 | 1.80 | 13.32 | 1.24 | 0.57 | 0.57 |
| 11 | 0.67 | 0.67 | 0.67 | 0.67 | 0.67 | 0.67 | 0.59 | 0.69 | 0.78 | 1.24 | 2.00 | 1.07 | 1.07 |
| 12 | 0.39 | 0.39 | 0.39 | 0.39 | 0.39 | 0.39 | 0.35 | 0.39 | 0.37 | 0.50 | 0.95 | 0.91 | 1.03 |
| 13 | 0.39 | 0.39 | 0.39 | 0.39 | 0.39 | 0.39 | 0.39 | 0.44 | 0.41 | 0.57 | 1.07 | 1.03 | 1.03 |

| Paraná |  |  |  |  |  |  |  |  |  |  |  |  |  |
| --- | --- | --- | --- | --- | --- | --- | --- | --- | --- | --- | --- | --- | --- |
| Age group # | **01** | **02** | **03** | **04** | **05** | **06** | **07** | **08** | **09** | **10** | **11** | **12** | **13** |
| 01 | 1.37 | 1.65 | 1.65 | 1.65 | 1.65 | 1.65 | 1.65 | 0.87 | 0.60 | 0.37 | 0.55 | 0.39 | 0.39 |
| 02 | 1.37 | 1.65 | 1.65 | 1.65 | 1.65 | 1.65 | 1.65 | 0.87 | 0.60 | 0.37 | 0.67 | 0.39 | 0.39 |
| 03 | 1.37 | 1.65 | 1.65 | 1.65 | 1.65 | 1.65 | 1.65 | 0.87 | 0.60 | 0.37 | 0.67 | 0.39 | 0.39 |
| 04 | 1.37 | 1.65 | 1.65 | 1.65 | 1.65 | 1.65 | 1.65 | 0.87 | 0.60 | 0.37 | 0.67 | 0.39 | 0.39 |
| 05 | 1.37 | 1.65 | 1.65 | 1.65 | 1.65 | 1.65 | 1.65 | 0.87 | 0.60 | 0.37 | 0.67 | 0.39 | 0.39 |
| 06 | 1.37 | 1.65 | 1.65 | 1.65 | 1.65 | 1.65 | 1.65 | 0.87 | 0.60 | 0.37 | 0.67 | 0.39 | 0.39 |
| 07 | 1.37 | 1.65 | 1.65 | 1.65 | 1.65 | 1.65 | 1.65 | 0.87 | 0.60 | 0.30 | 0.67 | 0.39 | 0.39 |
| 08 | 0.72 | 0.87 | 0.87 | 0.87 | 0.87 | 0.87 | 0.87 | 6.59 | 1.38 | 0.36 | 0.65 | 0.44 | 0.44 |
| 09 | 0.60 | 0.60 | 0.60 | 0.60 | 0.60 | 0.60 | 0.49 | 1.38 | 13.07 | 1.69 | 0.73 | 0.41 | 0.41 |
| 10 | 0.37 | 0.37 | 0.37 | 0.37 | 0.37 | 0.37 | 0.30 | 0.36 | 1.69 | 12.46 | 1.16 | 0.57 | 0.57 |
| 11 | 0.67 | 0.67 | 0.67 | 0.67 | 0.67 | 0.67 | 0.55 | 0.65 | 0.73 | 1.16 | 1.87 | 1.07 | 1.07 |
| 12 | 0.39 | 0.39 | 0.39 | 0.39 | 0.39 | 0.39 | 0.32 | 0.36 | 0.34 | 0.47 | 0.89 | 0.85 | 1.03 |
| 13 | 0.39 | 0.39 | 0.39 | 0.39 | 0.39 | 0.39 | 0.39 | 0.44 | 0.41 | 0.57 | 1.07 | 1.03 | 1.03 |

| São Paulo |  |  |  |  |  |  |  |  |  |  |  |  |  |
| --- | --- | --- | --- | --- | --- | --- | --- | --- | --- | --- | --- | --- | --- |
| Age group # | **01** | **02** | **03** | **04** | **05** | **06** | **07** | **08** | **09** | **10** | **11** | **12** | **13** |
| 01 | 1.41 | 1.65 | 1.65 | 1.65 | 1.65 | 1.65 | 1.65 | 0.87 | 0.60 | 0.37 | 0.57 | 0.39 | 0.39 |
| 02 | 1.41 | 1.65 | 1.65 | 1.65 | 1.65 | 1.65 | 1.65 | 0.87 | 0.60 | 0.37 | 0.67 | 0.39 | 0.39 |
| 03 | 1.41 | 1.65 | 1.65 | 1.65 | 1.65 | 1.65 | 1.65 | 0.87 | 0.60 | 0.37 | 0.67 | 0.39 | 0.39 |
| 04 | 1.41 | 1.65 | 1.65 | 1.65 | 1.65 | 1.65 | 1.65 | 0.87 | 0.60 | 0.37 | 0.67 | 0.39 | 0.39 |
| 05 | 1.41 | 1.65 | 1.65 | 1.65 | 1.65 | 1.65 | 1.65 | 0.87 | 0.60 | 0.37 | 0.67 | 0.39 | 0.39 |
| 06 | 1.41 | 1.65 | 1.65 | 1.65 | 1.65 | 1.65 | 1.65 | 0.87 | 0.60 | 0.37 | 0.67 | 0.39 | 0.39 |
| 07 | 1.41 | 1.65 | 1.65 | 1.65 | 1.65 | 1.65 | 1.65 | 0.87 | 0.60 | 0.31 | 0.67 | 0.39 | 0.39 |
| 08 | 0.74 | 0.87 | 0.87 | 0.87 | 0.87 | 0.87 | 0.87 | 6.82 | 1.43 | 0.37 | 0.67 | 0.44 | 0.44 |
| 09 | 0.60 | 0.60 | 0.60 | 0.60 | 0.60 | 0.60 | 0.51 | 1.43 | 13.52 | 1.74 | 0.76 | 0.41 | 0.41 |
| 10 | 0.37 | 0.37 | 0.37 | 0.37 | 0.37 | 0.37 | 0.31 | 0.37 | 1.74 | 12.89 | 1.20 | 0.57 | 0.57 |
| 11 | 0.67 | 0.67 | 0.67 | 0.67 | 0.67 | 0.67 | 0.57 | 0.67 | 0.76 | 1.20 | 1.93 | 1.07 | 1.07 |
| 12 | 0.39 | 0.39 | 0.39 | 0.39 | 0.39 | 0.39 | 0.34 | 0.37 | 0.35 | 0.49 | 0.92 | 0.88 | 1.03 |
| 13 | 0.39 | 0.39 | 0.39 | 0.39 | 0.39 | 0.39 | 0.39 | 0.44 | 0.41 | 0.57 | 1.07 | 1.03 | 1.03 |

**Table. Transmission probability per contact by age group [3].**

| Age group # | Age group | Transmission probability |
| --- | --- | --- |
| 01 | 0-1m | 8.0 * 10^-4^ |
| 02 | 2-3m | 8.0 * 10^-4^ |
| 03 | 4-5m | 8.0 * 10^-4^ |
| 04 | 6-8m | 8.0 * 10^-4^ |
| 05 | 9-11m | 8.0 * 10^-4^ |
| 06 | 12-23m | 9.0 * 10^-4^ |
| 07 | 2-4ys | 8.2 * 10^-4^ |
| 08 | 5-9ys | 8.6 * 10^-4^ |
| 09 | 10-14ys | 4.4 * 10^-4^ |
| 10 | 15-19ys | 1.6 * 10^-4^ |
| 11 | 20-49ys | 1.4 * 10^-4^ |
| 12 | 50-79ys | 0.05 * 10^-4^ |
| 13 | 80+ys | 1.0 * 10^-4^ |

**2. Demographic and epidemiologic data**

***Demographic data***

Brazilian population data files are available from the National population censuses (1980, 1991, 2000 and 2010), the population count (1996), and population estimates (for the remaining years) carried out by the Brazilian Institute of Geography and Statistics (IBGE, <http://www.ibge.gov.br>).

Total population counts all residents regardless of legal status or citizenship, except for refugees.

As IBGE does not provide population estimates disaggregated by geographical areas and age (for specific age in years or age groups) for all years, the Ministry of Health has carried out some estimations for the intercensal years using a standard methodology for the period of 1980 to 2012. These estimations have been done by the Department of Informatics of the Ministry of Health (DATASUS; http://www.datasus.gov.br) and are available in its website (<http://www2.datasus.gov.br/DATASUS/index.php?area=0206&id=6942&VObj=http://tabnet.datasus.gov.br/cgi/deftohtm.exe?ibge/cnv/pop>).

As in the DATASUS population data files there are no estimates for age in months for children up to one year of age, these were obtained for the 2000 and 2010 census years, from the IBGE website (<https://sidra.ibge.gov.br/pesquisa/censo-demografico/demografico-2010/inicial>). Interpolations were carried out to complete the gap years-months/age group.

The Ministry of Health set up the National Live Birth Information System (Sinasc) in 1994. Aggregated data were obtained from the government Internet site (<http://www2.datasus.gov.br/DATASUS/index.php?area=0205&id=6936&VObj=http://tabnet.datasus.gov.br/cgi/deftohtm.exe?sinasc/cnv/nv>). Data covers the period of 1994 to 2014. We calculated the average annual number of births for each state and used it for model input.

***Epidemiologic data***

*Outpatient cases*

Pertussis is a mandatory National Notifiable Disease since 1975. Initially, notifications were performed in standardized forms in an aggregated format. From 1998 onwards, electronic case-based reporting has been done into the National Information System for Notifiable Diseases (Sinan). Suspected cases are defined as any individual, regardless of age or vaccination status, with cough for at least 14 days, with either 1) one of the following symptoms: paroxystic cough, inspiratory stridor, post cough vomiting, cyanosis, apnea, or asphyxia, or 2) contact with confirmed case. Notified cases are investigated and can be confirmed by any of the following 3 criteria: (1) Lab: Culture or rtPCR confirmation (PCR implemented in 2010 only in selected states and for a percent of cases by the national reference laboratory). (2) Epidemiological link: Contact with lab confirmed case during infectious period. (3) Clinical: Presence of at least 2 pertussis related symptoms (the most stringent should be notified, Lab > Epi link > Clinical).

Outpatient cases data were obtained directly from the pertussis technical office of the Ministry of Health for the period of 1999 to 2016, nominal information (e.g. name and address of patients) was not made available. Outpatient cases were defined, based on the Brazilian surveillance system (SINAN, Sistema de Informação de Agravos de Notificação), as all confirmed cases minus confirmed cases that were hospitalized. Data was available by month and age group (17 age groups).

*Hospitalized cases*

Hospitalized cases were defined, based on the Brazilian hospital information system (SIH, Sistema de Informações Hospitalares do Sistema Único de Saúde). Data was available for years 1998 to 2016 by month and age group (13 age groups). Since this data represents hospitalizations occurring within the public health system, we adjusted the number of hospitalizations by SUS coverage to account for all possible hospitalizations. The assumption is that pertussis cases requiring hospitalization occurred at the same rate among those attended for in the public versus private system.

*Mortality data*

The Ministry of Health set up the National Mortality Information System (SIM) in 1976. ICD-9 coding was used to classify causes of death from 1976 to 1995, and ICD-10 has been used since 1996. One 3-digit code for pertussis in ICD-9 and ICD-10 implies that change in ICD coding should not impact pertussis mortality information.

Case based data were obtained directly from the Department of Health Information Analysis (DASIS) of the Ministry of Health for the period of 1979 to 2015. Nominal information (e.g. name and address of patients) was not made available. *Deaths* were taken as reported on the Brazilian mortality system (SIM, Sistema de Informação de Mortalidade). Data was available for years 1979 to 2016 by month and age group (17 age groups).

*Pertussis-specific mortality (state-specific)*

Pertussis-specific mortality was defined, for each state, as the number of deaths from pertussis as defined in the mortality data section above divided by the number of hospitalized cases after adjustment for SUS coverage.

**3. Vaccine parameters**

**3.1 Infant Vaccine Coverage**

Data on Infant pertussis vaccine coverage were drawn from two surveys conducted in Brazil, one in 1996 and one in 2007. The only data available for the three states came from the 2007 survey for the capitals of those states: Salvador for Bahia; Curitiba for Parana; Sao Paulo for Sao Paulo.

The National Health and Demography Survey, 1996 (Pesquisa Nacional Sobre Demografia e Saúde 1996. Sociedade Civil Bem-Estar Familiar no Brasil, BEMFAM, Programa de Pesquisas de Demografia e Saúde (DHS) Macro International Inc. March, 1997. Available at <https://dhsprogram.com/pubs/pdf/fr77/fr77.pdf>) is based on a representative sample of all 5 macro-regions in Brazil (South, Center-West, Northeast, Southeast, and South) and two major States (Rio de Janeiro and São Paulo). In the 1996 survey, the vaccination experience of children born since January 1991 and alive at survey (3,001 aged <2 years) was documented through home interviews conducted from March 1st through June 30th. Vaccine coverage was estimated based on the mother’s report and doses registered in the child’s immunization booklet.

The National Survey of vaccine coverage in urban state capitals in Brazil, 2007 (Barata et al 2012. Journal of Epidemiology and Community Health. 2012;66(10):934-941) is based on a representative sample of 26 state capitals and the Brasilia federal district, which account for 23.7% of total population. Between August 2007 and June 2008, the vaccination experience of children less than 18 months of age was documented. Vaccine coverage was estimated based on the mother’s report and doses registered in the child’s immunization booklet (available for 17,295 children).

For both surveys (1996 and 2007) we considered applied doses, irrespective of the timing of the first dose and time interval between doses. Using individual data on applied vaccine doses by children from these two surveys, Colin Sanderson provided us with vaccination coverage modeled by dose and week of age up to 3 years of age for 1996 and 2007. Coverage for each dose was cumulative so that the proportion of infants vaccinated rose monotonically with week of age. The methods are described in Clark A, Sanderson C. Timing of children’s vaccinations in 45 low-income and middle-income countries: an analysis of survey data. Lancet 2009; 373:1543–9 and the 1996 data were prepared for that study. Sanderson applied the same methods to the 2007 survey data, provided by the study principal investigator Dr José Cassio de Moraes. Vaccination coverage for the years between 1996 and 2007 was set by linear interpolation.

From Sanderson’s modeled data we calculated the probability that an infant in each of six age intervals (0-1, 2-3, 4-5, 6-8, 9-11, and 12-23 months) received a given dose. To represent not just coverage but protection the calculations used data for the midpoint of each age interval since a few weeks must elapse after a dose is given before the infant has developed immunity. The calculation of the probabilities is explained in general in the paragraphs below and for each probability in the tables that follow.

For the first dose, the numerator of the probability was the proportion of infants who received a first dose between the midpoint of the previous age interval and the midpoint of the age interval of interest. For example, for the age interval 4-5 months the numerator was the proportion of infants who received the first dose at the midpoint of the 4-5-month age interval, minus those who had received it at the midpoint of the 2-3-month age interval. The denominator was the proportion of infants who had not received a first dose as of the midpoint of the previous age interval. The denominator for the example was thus 1 minus the proportion of infants who had received the first dose at the midpoint of the 2-3-month age interval.

For doses 2 and 3 the numerator of the probability was the proportion of infants who received that dose between the midpoint of the previous age interval and the midpoint of the age interval of interest. The denominator was defined to take into account that the infant must have received dose 1 to be eligible for dose 2, or dose 2 to be eligible for dose 3, and, following the Brazilian schedule for infant vaccination, must have received that dose 2 months or more before the dose of interest. For the age intervals 2-3 months and 4-5 months, each exactly two months long, the denominator was thus the proportion of infants who had received the required dose, say dose 1, at the midpoint of the previous age interval minus those who had received the dose of interest, say dose 2, at the same time point. For the age intervals 6-8, 9-11, and 12-23 months, each longer than two months, the denominators were defined as the proportion of infants who had received the preceding dose two months before the midpoint of the age interval of interest. For example, the denominator for the probability that infants received dose 2 at 18 months was the proportion of infants who had received dose 1 by 16 months, two months earlier, minus the proportion who had received dose 1 at the midpoint of the previous age interval, 9-11 months, or, more specifically, the proportion who had received dose 1 by 69 weeks minus the proportion who had received dose 2 at 45 weeks.

To obtain standard errors for the probabilities, we used the formula: SQRT(p*(1-p))/N) where, p=proportion who received a given dose in a given age interval and N=number of infants in the survey.

For 2007, the probabilities calculated in this manner are shown below for the capitals of the 3 Brazilian states. For 1996, we used the ratio of the overall DPT coverage for Brazil relative to each state to adjust the probabilities estimated for Brazil as described (Pesquisa Nacional Sobre Demografia e Saúde 1996. Sociedade Civil Bem-Estar Familiar no Brasil, BEMFAM, Programa de Pesquisas de Demografia e Saúde (DHS) Macro International Inc. March, 1997. Available at <https://dhsprogram.com/pubs/pdf/fr77/fr77.pdf>). DPT vaccine coverage as estimated in the 1996 study was 80.8% for Brazil, 68.7% for the Northeast (where Bahia is located), 96.4% for the South (where Paraná is located), and 79.2% in São Paulo.

**Table. Conditional probability of being vaccinated by vaccine dose and age group in Brazil and the states, 1996.**

| Symbol | Description | Brazil | Bahia | Paraná | São Paulo |
| --- | --- | --- | --- | --- | --- |
| vc1 | Probability of receiving 1^st^ dose |  |  |  |  |
|  | At age [2-3) months | 0.699588 | 0.5948 | 0.8347 | 0.6857 |
|  | At age [4-5) months | 0.563578 | 0.4792 | 0.6724 | 0.5524 |
|  | At age [6-8) months | 0.298626 | 0.2539 | 0.3563 | 0.2927 |
|  | At age [9-11) months | 0.220444 | 0.1874 | 0.2630 | 0.2161 |
|  | At age [12-23) months | 0.263642 | 0.2242 | 0.3145 | 0.2584 |
| vc2 | Probability of receiving 2^nd^ dose |  |  |  |  |
|  | At age [4-5) months | 0.810366 | 0.6890 | 0.9668 | 0.7943 |
|  | At age [6-8) months | 0.748350 | 0.6363 | 0.8928 | 0.7335 |
|  | At age [9-11) months | 0.529659 | 0.4503 | 0.6319 | 0.5192 |
|  | At age [12-23) months | 0.586053 | 0.4983 | 0.6992 | 0.5744 |
| vc3 | Probability of receiving 3^rd^ dose |  |  |  |  |
|  | At age [6-8) months | 0.821852 | 0.6988 | 0.9805 | 0.8056 |
|  | At age [9-11) months | 0.611181 | 0.5197 | 0.7292 | 0.5991 |
|  | At age [12-23) months | 0.562642 | 0.4784 | 0.6713 | 0.5515 |

**Table. Conditional probability of being vaccinated by vaccine dose and age group in Bahia, Paraná and São Paulo, 2007.**

| Symbol | Description | Salvador (Bahia) | Curitiba (Paraná) | São Paulo (São Paulo) |
| --- | --- | --- | --- | --- |
| vc1 | Probability of receiving 1^st^ dose |  |  |  |
|  | At age [2-3) months | 0.292943 | 0.324054 | 0.366795 |
|  | At age [4-5) months | 0.291902 | 0.250412 | 0.288618 |
|  | At age [6-8) months | 0.401596 | 0.417582 | 0.425714 |
|  | At age [9-11) months | 0.528889 | 0.467924 | 0.616915 |
|  | At age [12-23) months | 0.754717 | 0.624114 | 0.987013 |
| vc2 | Probability of receiving 2^nd^ dose |  |  |  |
|  | At age [4-5) months | 0.700000 | 0.731959 | 0.733333 |
|  | At age [6-8) months | 0.725410 | 0.736641 | 0.730290 |
|  | At age [9-11) months | 0.807229 | 0.896296 | 0.882845 |
|  | At age [12-23) months | 0.930481 | 0.948454 | 0.966102 |
| vc3 | Probability of receiving 3^rd^ dose |  |  |  |
|  | At age [6-8) months | 0.637838 | 0.646341 | 0.720165 |
|  | At age [9-11) months | 0.798561 | 0.782334 | 0.785965 |
|  | At age [12-23) months | 0.945245 | 0.931707 | 0.931064 |

**3.2 Maternal vaccine coverage**

In Brazil, maternal Tadp vaccine implementation began in 2014. For each state, we used Tadp vaccine coverage was observed for that state (Bahia: 12.3% in 2014 and 43.0% in 2015; Paraná: 6.1% in 2014 and 30.8% in 2015; São Paulo: 12.5% in 2014 and 61.1% in 2015) and then assumed that vaccine coverage would linearly increase each year and reach 70% of women in 20130.

**3.3 Vaccine Efficacy**

Maternal aP vaccine efficacy. The estimate of maternal aP vaccination effectiveness comes from an English study [Amirthalingam G, Andrews N, Campbell H, Ribeiro S, Kara E, Donegan K, Fry NK, Miller E, Ramsay M. Effectiveness of maternal pertussis vaccination in England: An observational study. Lancet 2014; 384: 1521-28. PMID: 25037990], which found that maternal TdaP vaccine effectiveness in infants younger than 3 months was 91% (95% CI: 84 - 95). We assume that maternal immunization protects infants for the first 3-4 months of life.

Infant vaccine efficacy. The efficacy of routine infant vaccine by dose was taken from Juretzko et al. [Juretzko P, von Kries R, Hermann M, Wirsing von König CH, Weil J, Giani G. Effectiveness of acellular pertussis vaccine assessed by hospital-based active surveillance in Germany. Clin Infect Dis. 2002 Jul 15;35(2):162-7. PMID: 12087522 DOI: 10.1086/341027], the only study we found that reports efficacy by dose (shown as proportions in the table below). We did not adjust the vaccine efficacy of DTP1 for any possible decrease or increase in protection from maternal immunization.

**Table. Vaccine efficacy by dose of infant vaccination.**

| ve1 | wP, 1^st^ dose | 0.68 (95%CI: 0.456 - 0.811) |
| --- | --- | --- |
| ve2 | wP, 2^nd^ dose | 0.92 (95% CI: 0.847 - 0.957) |
| ve3 | wP, 3^rd^ dose | 0.99 (95% CI: 0.989 - 1.000) |

Although Juretzko evaluated an aP vaccine and the models assume a wP vaccine, a 2016 systematic review of efficacy of both vaccine types (aP and wP) in children [14] shows that Juretzko’s results closely resemble those for wP vaccines. The review found that 3 or more doses of wP vaccine were 94% effective against pertussis, compared with 84% for 3 or more doses of aP vaccine.

**4. Cost parameters**

All costs are reported as 2014 US$. Except for vaccine purchase costs, which are already in dollars for international purchase through the Pan American Health Organization’s Revolving Fund, all costs were estimated in Brazilian Reals (BRL) and converted to 2014 US dollars using the official Brazilian Central Bank Exchange Rate on Dec 31, 2014 (<http://www4.bcb.gov.br/pec/conversao>) when 1 BRL = 0.43 USD.

- 1. **Costs of vaccination**

Maternal vaccine price/dose. Brazil began maternal aP immunization in 2014 using a single dose vaccine of multivalent formulation (adolescent/adult TdaP) purchased through the Pan American Health Organization Revolving Fund. Data was obtained from the Pan American Health Organization Revolving Fund 2014 price list (Source: [http://www.**paho**.org](http://www.paho.org)).

To the base price per dose (USD 8.905), freight and insurance charges (3%) and a service charge (4.25%) were added, bringing the total to USD 9.55. Further adding an additional 5% wastage rate, as recommended by WHO, brings the cost for each vaccine dose to **USD 10.028. In sensitivity analysis we varied the wastage rate from 0-15%.**

Incremental maternal delivery cost/dose. Delivery cost has not been separately estimated for maternal aP immunization. In Brazil, since Td is being replaced by TdaP, it is assumed that there is no incremental cost of delivering TdaP.

Infant vaccine price/dose. We used the 2014 listed dose price for the single dose pentavalent vaccine (DTP-HepB-Hib) liquid formulation, which is purchased through the Pan American Health Organization Revolving Fund (Source: [http://www.**paho**.org](http://www.paho.org)). To the base price per dose (USD 2.5251), freight and insurance charges (3%) and a service charge (4.25%) were added, totaling USD 2.708. Further adding an additional 5% wastage rate, as recommended by WHO, would bring the cost for each vaccine dose to **USD 2.84**. **In sensitivity analysis we varied the wastage rate from 0-15%.**

Vaccine delivery cost/dose (for any age group). The cost of delivering a dose of vaccine for any individual was obtained from a primary study conducted in 2013 in Brazil which estimated the cost of the immunization program in the country (Toscano et al., manuscript under review). **In sensitivity analysis we varied the delivery cost within its 95% confidence interval.**

- 1. **Costs of illness**

Costs for pertussis outpatient cases

The cost of outpatient management of pertussis was estimated based on standardized guidelines for pertussis case management from the Brazilian Ministry of Health and Tertiary care Reference Hospitals in the country [Ministério da Saúde 2009. Guia de Vigilância Epidemiológica, Série A: Normas e Manuais Técnicos. 819 p. Available from: <http://bvsms.saude.gov.br/bvs/publicacoes/guia_vigilancia_epidemiologica_7ed.pdf>. Ministério da Saúde 2017. Guia de Vigilância em Saúde. Secretaria de Vigilância em Saúde. Coordenação-Geral de Desenvolvimento da Epidemiologia em Serviços. Sociedade Brasileira de Pediatria 2013. Coqueluche: Recomendações atuais. <http://www.sbp.com.br/imprensa/detalhe/nid/coquelu>]. These cost estimates apply to patients whose pertussis does not require hospital care. Costs for hospitalized patients were estimated separately; see below. In sensitivity analysis we varied the cost for pertussis outpatient cases using an upper value that considers antibiotic treatment, as detailed in the following.

Direct medical costs were estimated considering the following cost components, which are the healthcare resources used for the outpatient management of a pertussis patient: Diagnostic exams, medical visits, and medications.

Diagnostic exams included specific pertussis diagnosis (bacterial isolation, considered the gold standard for diagnosis), and complementary exams (chest X-ray for children < 5 years of age and blood cell count for all patients). Two medical visits were assumed for each case. Medications included antipyretic (paracetamol 200mg/mL, 15 mL) and Azithromycin as the first-line antibiotic, as recommended by the guidelines.

For diagnosis and medical visits, unit costs were obtained from the standardized national pricing lists of the Brazilian Public Healthcare System (Sistema de Gerenciamento da Tabela de Procedimentos, Medicamentos e OPM do SUS -(SIGTAP/DATASUS), as depicted in Table 1 below [Ministério da Saúde. Sistema de Gerenciamento da Tabela de Procedimentos, Medicamentos e OPM do SUS 2014. Available from: <http://sigtap.datasus.gov.br/tabela-unificada/app/sec/inicio.jsp>]. Unit costs were then multiplied by quantities to obtain the estimated cost per case of a patient without complications, managed as an outpatient.

**Table. Unit costs for pertussis diagnosis and management, 2014 (in Brazilian Reais, R$)**

| **Items** | **Code in SIGTAP** | **Unit cost in BRL** | **Total costs** |
| --- | --- | --- | --- |
| Thorax X-ray (AP and lateral) | 02.04.03.015-3 | R$ 9.50 | R$ 9.50 |
| Blood cell count | 02.02.02.039-8 | R$ 2.73 | R$ 2.73 |
| Cultures for pertussis identification | 02.02.08.008-0 | R$ 5.62 | R$ 5.62 |
| Medical visit | 03.01.01.007-2 | R$ 10.00 | R$ 20.00 |

| **Sub-total costs** | Individuals <5 years of age | R$ 37.85 |
| --- | --- | --- |
|  | Individuals aged 5+ years | R$ 28.35 |

For medications, the weighted average of government purchases of a given medication in the 12 months January/2014 - December/2014, according to the National Price Bank [Banco de preços em saúde, 2014. Available from: <http://portalms.saude.gov.br/gestao-do-sus/economia-da-saude/banco-de-precos-em-saude>] were used. In the base case we considered Azithromycin as the first-line antimicrobial agent, and in sensitivity analysis, we considered Clarithromycin (which is the alternative recommended antimicrobial agent with higher costs). In addition, paracetamol use for 4 days was considered in the following dosage: 1) <8 years, 1 drop/kg; 3 times/day, 2) 8 years and older 40 drops; 3 times/day. Azithromycin was considered in both powder for oral suspension (600mg) for children <13 years old, or 250mg or 500mg capsules depending on recommended dose by weight. The following age groups were considered: 0-1m, 2-3m, 4-5m, 6-8m, 9-11m, 12-23m, 2-4 years, 5-9 years, 10-14 years, 15-19 years, and 20 years and older (adults). Average body weights for each age group by gender were estimated based on standardized WHO Z-scores curves for weight and height [The WHO Child Growth Standards, 2007. Available from: <http://www.who.int/childgrowth/standards/weight_for_age/en/>]. Total cost by cost component and by age group is presented below in the Table.

**Table. Costs for outpatient pertussis case management, by age group, in Brazilian Reais (R$)***

| Age group | Diagnosis | Medical visit | Medications | Total Cost | Second line antibiotics* | Total Cost* |
| --- | --- | --- | --- | --- | --- | --- |
| < 3 years | R$18.15 | R$20.00 | R$2.21 + R$0.54 | R$40.90 | R$ 26.30+R$0.54 | R$64.99 |
| 3-4 years | R$18.15 | R$20.00 | R$2.21 + R$0.54 | R$40.90 | R$ 52.60+R$0.54 | R$91.29 |
| 5 years | R$8.35 | R$20.00 | R$2.21 +R$0.54 | R$31.10 | R$ 52.60+R$0.54 | R$81.49 |
| 6 years | R$8.35 | R$20.00 | R$2.21 +R$0.54 | R$31.10 | R$ 52.60+R$0.54 | R$81.49 |
| 7 years | R$8.35 | R$20.00 | R$2.21 +R$0.54 | R$31.10 | R$ 52.60+R$0.54 | R$81.49 |
| 8 years | R$8.35 | R$20.00 | R$2.21 +R$0.54x2 | R$31.64 | R$ 52.60+R$0.54x2 | R$82.03 |
| 9 years | R$8.35 | R$20.00 | R$2.21 +R$0.54x2 | R$31.64 | R$ 52.60+R$0.54x2 | R$82.03 |
| 10-11 years | R$8.35 | R$20.00 | R$2.21 +R$0.54x2 | R$31.64 | R$ 76.64+R$0.54x2 | R$106.07 |
| 10-12 years | R$8.35 | R$20.00 | R$4.42 +R$0.54x2 | R$33.85 | R$ 76.64+R$0.54x2 | R$106.07 |
| 13 years | R$8.35 | R$20.00 | R$6.63 +R$0.54x2 | R$36.06 | R$ 76.64+R$0.54x2 | R$106.07 |
| 14 years | R$8.35 | R$20.00 | R$10.66+R$0.54x2 | R$40.09 | R$76.64+R$0.54x2 | R$106.07 |
| 15-19 years | R$8.35 | R$20.00 | R$10.66+R$0.54x2 | R$40.09 | R$76.64+R$0.54x2 | R$106.07 |
| Adults | R$8.35 | R$20.00 | R$10.66+R$0.54x2 | R$40.09 | R$50.32+R$0.54x2 | R$79.75 |

*Considered in sensitivity analysis of outpatient costs.

Costs estimated in 2014 Brazilian Reais and converted to US dollars considering the 2014 official exchange rate where R$1.00=0.43USD in 2014.

Costs for hospitalized pertussis patients, by survived or died

The average costs of inpatient treatment for pertussis cases, stratified by age sub-group and by patient outcome (alive at the end of hospitalization or died during hospitalization), and their standard errors, were obtained from reimbursements paid for all pertussis cases hospitalized in 2014 in the hospitals of the Brazilian National Public Health system (SUS), which covers 75% of the Brazilian population. **In sensitivity analysis we varied the inpatient cost within its 95% confidence interval (+/-1.96*standard error).**

Reimbursements include direct medical and non-medical costs. Direct medical costs include hospital stay, healthcare professional services, and physical therapy. Non-medical costs include the stay of a parent or caregiver accompanying the hospitalized child, which is paid for by SUS to the hospital.

Reimbursements for each cost item are standardized nationwide within SUS (SIGTAP) (<http://sigtap.datasus.gov.br/tabela-unificada/app/sec/procedimento/exibir/0303010037/02/2014>). The hospital stay is valued based on a standard stay by ICD10 diagnostic code (A37 for pertussis). Pertussis reimbursement is BRL 793.69 for a hospital stay of up to 12 days, after which an additional BRL 20.00 per day is paid. The standard reimbursement for healthcare professional services for pertussis is BRL 72.22, which may increase depending on the need for additional specialty professionals. Each physical therapy session is an additional BRL 6.35. Each day of hospital stay of an accompanying parent or caregiver is reimbursed at BRL 8.00.

Costs are further stratified by patient outcome (dead or alive at the end of hospitalization). Average costs, standard error, standard deviation, and minimum and maximum observed reimbursement values are reported for each strata. Highlighted in yellow are strata for which no cases and thus no reimbursement values were obtained. To generate estimates for these strata, data from the other age groups were linearly interpolated.

**Table. Costs for hospitalized pertussis case, by outcome and age group, in Brazilian Reais (R$)***

| Age group and outcome | | Mean (R$) | N | SD | SE | Minimum | Maximum |
| --- | --- | --- | --- | --- | --- | --- | --- |
| 0-1m | Alive | 1644,09 | 1554 | 2509,22 | 63,65 | 47,27 | 29420,27 |
|  | Died | 2647,04 | 30 | 2391,44 | 436,61 | 865,91 | 11788,61 |
|  | Total | 1663,08 | 1584 | 2510,04 | 63,07 | 47,27 | 29420,27 |
| 2-3m | Alive | 1264,50 | 1672 | 1839,51 | 44,99 | 44,22 | 29576,04 |
|  | Died | 3362,82 | 14 | 3386,80 | 905,16 | 881,91 | 13310,06 |
|  | Total | 1281,93 | 1686 | 1865,60 | 45,43 | 44,22 | 29576,04 |
| 4-5m | Alive | 1094,51 | 677 | 1006,60 | 38,69 | 47,27 | 15517,05 |
|  | Died | 2365,92 | 3 | 1416,03 | 817,55 | 960,40 | 3792,23 |
|  | Total | 1100,12 | 680 | 1010,83 | 38,76 | 47,27 | 15517,05 |
| 6-8m | Alive | 1144,54 | 286 | 1622,76 | 95,96 | 47,27 | 21291,11 |
|  | Total | 1144,54 | 286 | 1622,76 | 95,96 | 47,27 | 21291,11 |
| 9-11m | Alive | 952,20 | 108 | 314,09 | 30,22 | 47,27 | 1947,67 |
|  | Died | 15840,58 | 1 | NA | NA | 15840,58 | 15840,58 |
|  | Total | 1088,79 | 109 | 1459,92 | 139,83 | 47,27 | 15840,58 |
| 12-23m | Alive | 1141,72 | 146 | 1715,83 | 142,00 | 47,27 | 19478,43 |
|  | Died | 1207,62 | 3 | 421,33 | 243,25 | 873,91 | 1681,06 |
|  | Total | 1143,05 | 149 | 1699,08 | 139,19 | 47,27 | 19478,43 |
| 2-4a | Alive | 961,13 | 208 | 593,24 | 41,13 | 47,27 | 6448,15 |
|  | Died | 3738,22 | 1 | NA | NA | 3738,22 | 3738,22 |
|  | Total | 974,42 | 209 | 622,21 | 43,04 | 47,27 | 6448,15 |
| 5-9a | Alive | 944,77 | 122 | 291,42 | 26,38 | 47,27 | 3392,86 |
|  | Total | 944,77 | 122 | 291,42 | 26,38 | 47,27 | 3392,86 |
| 10-17a | Alive | 913,85 | 46 | 244,60 | 36,06 | 47,27 | 1845,98 |
|  | Died | 6622,73 | 1 | NA | NA | 6622,73 | 6622,73 |
|  | Total | 1035,31 | 47 | 867,16 | 126,49 | 47,27 | 6622,73 |
| 18-39a | Alive | 913,79 | 25 | 124,77 | 24,95 | 865,91 | 1428,98 |
|  | Total | 913,79 | 25 | 124,77 | 24,95 | 865,91 | 1428,98 |
| 40-64a | Alive | 1049,89 | 13 | 278,63 | 77,28 | 865,91 | 1612,12 |
|  | Died | 1797,70 | 4 | 1863,58 | 931,79 | 865,91 | 4593,07 |
|  | Total | 1225,85 | 17 | 903,50 | 219,13 | 865,91 | 4593,07 |
| 65+ys | Alive | 2026,42 | 13 | 3592,75 | 996,45 | 52,22 | 13813,63 |
|  | Died | 1382,90 | 4 | 870,62 | 435,31 | 869,91 | 2685,81 |
|  | Total | 1875,00 | 17 | 3146,77 | 763,20 | 52,22 | 13813,63 |
| Total | Alive | 1319,72 | 4870 | 1914,22 | 27,43 | 44,22 | 29576,04 |
|  | Died | 2887,46 | 61 | 3006,85 | 384,99 | 865,91 | 15840,58 |
|  | Total | 1339,12 | 4931 | 1938,81 | 27,61 | 44,22 | 29576,04 |

SD: standard deviation. SE: standard error of the mean.

**5. Model output**

***Model output***

Model output include the number of outpatient and inpatient cases and the number of deaths, all which were used for the calculation of disability adjusted life years (DALYs) from pertussis. DALYs capture both premature death and disability within a single metric: it simultaneously measures the impacts of mortality and morbidity from a given health condition using time as the common metric. One DALY represents a year of healthy life lost and is calculated as the sum of two components: mortality, represented by the years of life lost due to premature death (YLL), and morbidity, the years lost due to disability (YLD).

For each case of pertussis, we calculated the disability imposed by it using the disability weight (D=1 for premature death, D=0 for perfect health, so smaller weights indicate less disability on an individual’s life). Duration of disability imposed by pertussis is given the duration of symptoms, assumed of 14 days. Disability weight for pertussis (0.051, range 0.032 - 0.074) was obtained from the Global Burden of Disease (GBD) Study website, operated by the Institute of Health and Metrics and Evaluation (IHME). The value is not age-specific. Source: Global Burden of Disease Collaborative Network. Global Burden of Disease Study 2016 (GBD 2016) Disability Weights. Seattle, United States: Institute for Health Metrics and Evaluation (IHME), 2017.

http://ghdx.healthdata.org/record/global-burden-disease-study-2016-gbd-2016-disability-weights

Years of life lost due to pertussis were calculated as the product of the number of deaths and the estimated

life expectancy for the age at which the death occurred. Following the methodology adopted in prior Burden of Disease studies from Brazil, we assumed life expectancies corresponding to 81.75 years (obtained as the mean of 80 years for men and 82.5 years for women). This standard was conceived as the highest attainable life expectancy when DALY was proposed, and its use allows the comparison of results [15]. Finally, we followed the most recent approach used in the 2010 Global Burden of Disease study and in the Brazilian study and did not use age-weights [16, 17].

**6. Model fitting procedures and results**

An important step towards proving the credibility and usefulness of a model is the process of model fitting or calibration. Model fitting included the calibration of model predictions to the observed data (calibration targets) in order to1) select the best-fitting model and 2) determine the values of the highly uncertain parameters. The highly uncertain parameters were: multiplier of infection probability per contact (for age groups: <1, 1-9, 10+ years), waning of immunity from infection and from infant vaccination, and duration of infection. Additionally, as cases are significantly under-reported, we explicitly incorporated a parameter for the reporting probability for symptomatic cases. In Section #1, we highlighted how the reporting probability aims to map model predicted infections to symptomatic cases. In the following paragraphs we describe the calibration targets, the model predictions, and the methodology.

*Calibration targets:* In the selection of the calibration targets, we searched for the best available good-quality data to use. As described in Section #2, we obtained monthly, age-specific outpatient, inpatient and mortality data from official sources for each of the modelled states for the period of 2000-2016. These three time series (manuscript, Figures 1, 2, and 3) show a clear pattern of very low incidence up to 2010 and a sharp increase in incidence between 2010 and 2014, as other epidemiologic studies from several regions in Brazil have shown [10, 11, 18]. The fact that the number of inpatient cases surpasses the number of outpatient cases in those <1 year of age likely reflects the much higher likelihood of severe disease in this age group.

We first attempted to use as calibration targets the age-specific outpatient, inpatient and mortality data for each Brazilian state. However, low incidence periods such as those observed for the years 2000-2010 makes the process of model fitting a challenge. Indeed, prior studies that have attempted to fit models to periods of low incidence (resulting from high vaccine coverage) have shown that the performance of the process significantly declines because the models are unable (or became unstable) to capture the transition to very low incidence [4]. Accordingly, model fitting to the time series as shown in Figures 1, 2, and 3 was not feasible due to low numbers. To reduce the computational burden, we aggregated, for each Brazilian state (São Paulo, Paraná, Bahia), the observed monthly age-specific outpatient, inpatient and mortality data into one annual age-specific time series of cases. Additionally, we aggregated the thirteen age-specific time series of cases into three broader age groups: <1 year, 1-9 years and 10+ years of age. Thus, for each Brazilian state (São Paulo, Paraná, Bahia), we used as calibration targets the annual number of cases (outpatient, inpatient, and deaths) for age groups <1, 1-9 years, and 10+ years.

*Model predictions:* These calibration targets were fitted to model predicted age-specific time series of infections. For both the SIR and the SIRS model the model predicted time series of infections included, by definition, only primary infections. For the SIRSI model, model predicted time series of infections included both primary and secondary infections. Model predicted age-specific time series of infections were grouped into the corresponding age groups: <1 year, 1-9 years and 10+ years of age, for each Brazilian state (São Paulo, Paraná, Bahia).

A summary of model fitting objectives, calibration targets and model predictions is provided below:

| **Objectives** | **Calibration targets** | **Model predictions** |
| --- | --- | --- |
| 1) Select the best-fitting model and 2) determine the values of the highly uncertain parameters: multiplier of infection probability per contact (for age groups: <1, 1-9, 10+ years), waning of immunity from infection and from infant vaccination, duration of infection, and reporting probability for symptomatic cases | Observed annual number of symptomatic cases (sum of outpatient, inpatient, and deaths) for age groups <1, 1-9 years, and 10+ years, for each Brazilian state (São Paulo, Paraná, Bahia) | Model predicted annual number of infections for age groups: <1 year, 1-9 years and 10+ years of age, for each Brazilian state (São Paulo, Paraná, Bahia) |

*Methodology:* Qualitative and quantitative assessment of the model fitting results were carried out to determine the best fitting set of parameters and model structure. Qualitative assessment focused on graphical displays of the predicted dynamics of pertussis incidence which should correspond to the observed data with respect to a dramatic decrease in incidence in the 1980/90s and a resurgence around 2010. Quantitative assessment focused on multiple goodness-of-fit (GOF) statistics including the mean absolute error (MAE), which measures the average magnitude of the errors in a set of predictions; the root mean square error (RMSE), which gives the standard deviation of the prediction error; the normalized root mean square error (NRMSE), given in percentage; and the ratio of the RMSE to the standard deviation of the observations (RSR). For all these measures, the lower the value, the better the fit.

The model fitting procedures included a combination of three approaches: assignment of parameter values based on previous or expert knowledge, parameter estimation based on inverse modelling via non-linear optimization approach that minimizes a least square function of the residuals, and parameter identifiability analysis by investigation of collinearity among parameter sets.

The estimation of the reporting probability for symptomatic cases required one extra step: the fitting of a functional regression model relating the observed data and the model predicted time series of infections. We approached the problem via Functional Data Analysis [19]. More specifically, we relied on regressing a “functional response” onto “functional covariates”. Function-on-function (fof) regression via model-based boosting is described in [20]. In this application of the technique, our functional response is given by the calibration targets (annual number of symptomatic cases for age groups <1, 1-9 years, and 10+ years, for each Brazilian state) and the functional covariate is given by the corresponding model predicted time series of infections, i.e., that predicted by the SIR, SIRS, and SIRSI models (model predicted annual number of infections for age groups: <1 year, 1-9 years and 10+ years of age, for each Brazilian state). Resulting from this regression is a second predicted time series for the observed data (let’s call it “fofPredicted”) for each corresponding combination of observed and model-specific predicted time series (that is, for each of the model structures, SIR, SIRS, and SIRSI). We then calculated the ratio of fofPredicted to the model-specific time series, which is itself a time series. The reporting probability is the mean value of this time series. Library “FDboost” was used for the fitting of the functional regression models (<https://arxiv.org/abs/1705.10662>).

The sensitivity analysis of key parameter values was carried out using Latin hypercube sampling available in function “sensFun” (library “FME” [<http://www.jstatsoft.org/v33/i03>]). This procedure consists in allowing certain parameters to vary within specified ranges reflecting the uncertainty of the estimates.

*Results*

All three model structures produce similar fit to the data. Results of the quantitative assessment of fit as estimated by each statistic is given in the main text (Table 3). We elected the SIRSI model for the final model structure based on the goodness of fit statistics and also on recent literature attesting the occurrence of pertussis infection in adolescents and adults as well as previously vaccinated children in Brazil [12, 21, 22].

The values of the highly uncertain parameters for the SIRSI model are as follows:

Estimates for the reporting probability varied by state: Bahia: mean 1.4% and range 0.2-5.2%, Paraná: mean 2.5% and range 0.4-7.9%, São Paulo: mean 3.4% and range 1.2-10.4%. It is important to note that fact that the reporting probability varies by state being highest in São Paulo and lowest in Bahia concurs with the resources and capacities in these settings [11, 18].

As for the other parameters, 10-fold multiplier of infection probability per contact for each age group (<1, 1-9, 10+) provided adequate fit. The values obtained for the duration of immunity were as follows: 25 years for infection and 14.5 years for each dose of infant Tdp vaccine (ranges explored in the sensitivity analysis included 10 to 50 years for immunity from infection and 5-25 years for immunity from Tdp vaccine). Finally, best fit model results for duration of infection was somewhat longer (25 days) though within the range assumed in prior analyses [23].

The following pages provide figures for the observed (filled circles and full lines) and predicted (empty circles and dotted lines) time series of reported cases in <1 year (black), 1-9 years (blue) and >10 years (purple) of age by model structure: SIR, SIRS and SIRSIs, in each state: São Paulo, Paraná, and Bahia.

**Sao Paulo**


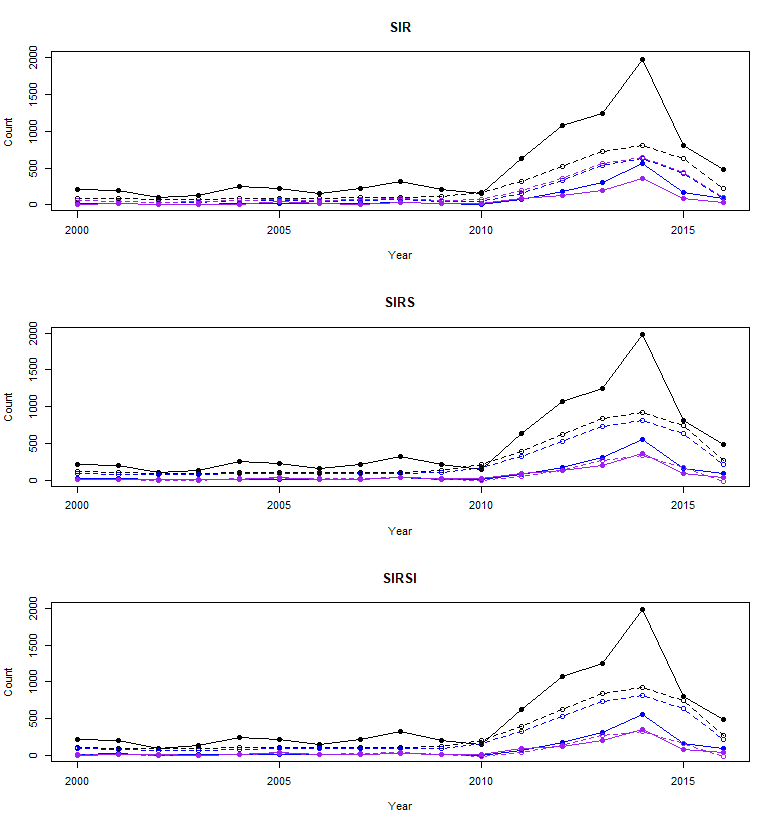


**Parana**


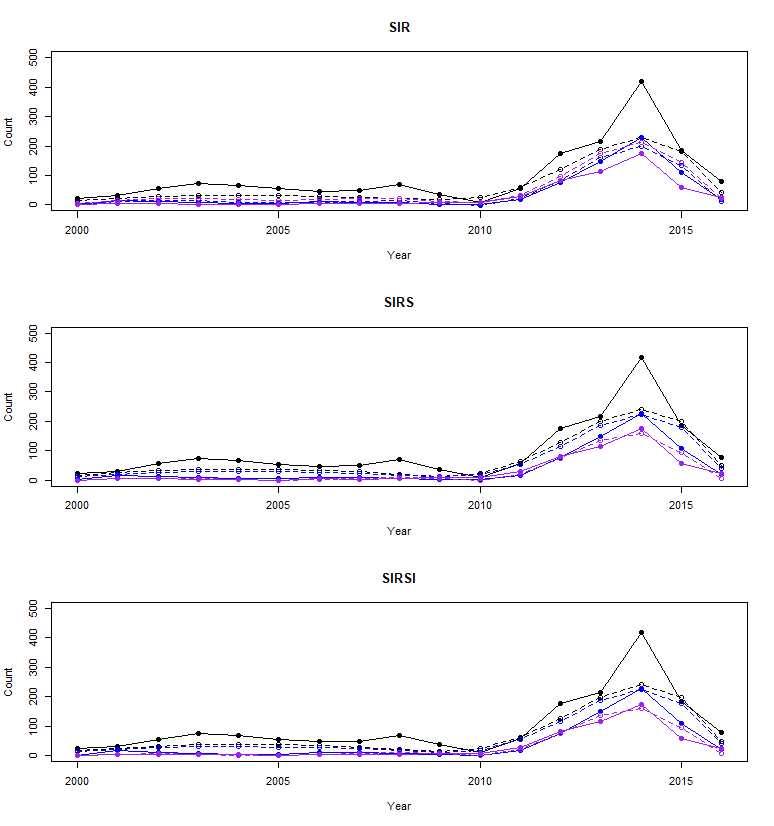


**Bahia**


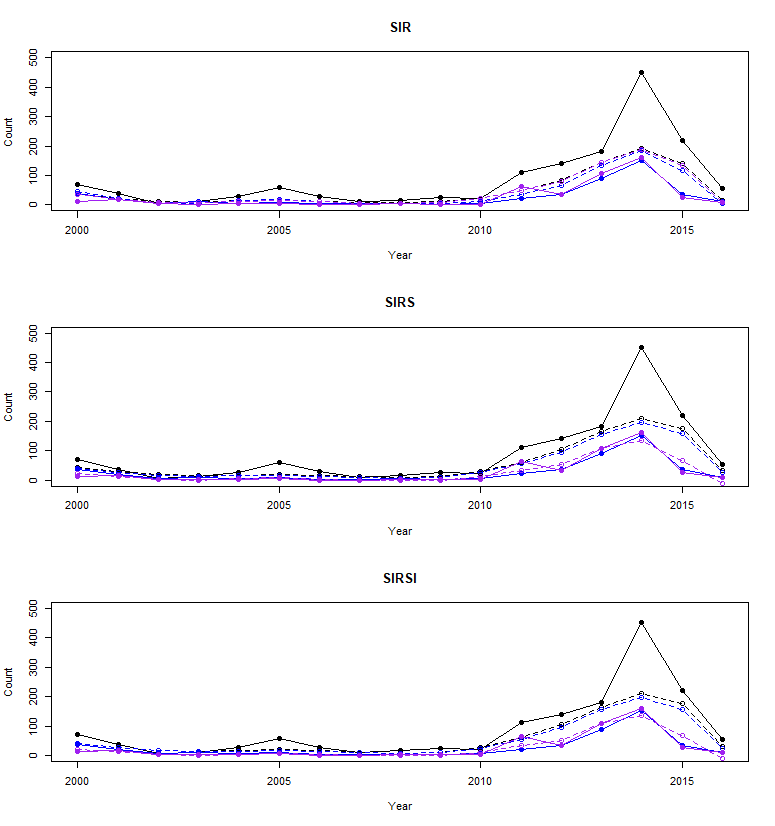


**7. Analysis**

**Interventions**

We examined two interventions: standard of care in Brazil prior to 2014 when no maternal immunization was provided (SOC) and maternal aP immunization.

**Outcomes**

Key outcomes included morbidity (outpatient and inpatient cases), mortality, years of life lost and disability adjusted life years lost due to pertussis in infants (<1 year of age), children (1-9 years of age) and adolescents/adults (10+ years).

**Discounting**

Health, costs, and cost-effectiveness outcomes were discounted at a 3% rate for base-case results. In sensitivity analyses, we varied the discount rate from 0% to 6%.

**Time horizon and perspective**

The impact of each strategy was simulated over a time horizon of 16 years starting from 2014 to 2029 and was evaluated from the perspective of the Brazilian National Health System.

**Cost-effectiveness**

By dividing the cost difference of intervention and comparator by the effect difference of intervention and comparator the results can be expressed as an incremental cost-effectiveness ratio (ICER), denominated in dollar costs per DALY averted. The ICER provides us with a measure of value for money. This can facilitate comparisons with alternative health care investments. The decision to switch to the new intervention can be evaluated by comparing the ICER to estimates of the societal willingness-to-pay for additional health gains. We defined an intervention to be cost-effective if its cost-effectiveness ratio, in terms of cost per DALY averted, was less than the country’s per capita Gross Domestic Product (GDP). According to the International Monetary Fund, Brazil’s 2014 GDP per capita is US$ 12,026. Any new strategy with an ICER below the willingness-to-pay threshold is considered to be cost-effective and should be adopted on the basis of representing better value for money than the current strategy.

**8. Symbols and notation**

| Symbol | Description | Notes |
| --- | --- | --- |
| Compartments |  |  |
| Demography |  |  |
| Age group # | Age group |  |
| 01 | 0-1m |  |
| 02 | 2-3m |  |
| 03 | 4-5m |  |
| 04 | 6-8m |  |
| 05 | 9-11m |  |
| 06 | 12-23m |  |
| 07 | 2-4ys |  |
| 08 | 5-9ys |  |
| 09 | 10-14ys |  |
| 10 | 15-19ys |  |
| 11 | 20-49ys |  |
| 12 | 50-79ys |  |
| 13 | 80+ys |  |
| Natural history |  |  |
| S | Susceptible to infection |  |
| I | Infected |  |
| R | Recovered | Immunity wanes in models SIRS and SIRSI |
| Ss | Susceptible to secondary infections | Applicable to model SIRSI |
| Is | Infected with secondary infections | Applicable to model SIRSI |
| Vaccine related |  |  |
| Vmp | Maternally vaccinated, protected |  |
| Vmn | Maternally vaccinated, not protected |  |
| V1p | Received V1, protected |  |
| V1n | Received V1, not protected |  |
| V2p | Received V2, protected |  |
| V2n | Received V2, not protected |  |
| V3p | Received V3, protected |  |
| V3n | Received V3, not protected |  |
| Parameters |  |  |
| Demography |  |  |
| Births | Births | # of births/day, state data from the Brazilian National Institute of Geography and Statistics. See Technical Appendix, Section #1. |
| di | Ageing | 1/duration of age group. For example, for the age group 10-14 years, duration is 5 years. Defined for each age group, i=1 to 12; there is no ageing parameter for the last age group. |
| mi | Mortality | Mortality rate from all causes estimated using state-specific population data from the National Institute of Geography and Statistics and all-cause mortality data from the National Mortality System. See Technical Appendix, Section #1. Defined for each age group, i=1 to 13. |
| Natural history | |  |
| fi | Force of infection | Time-dependent parameter based on age-specific contact rates and the pool of infectious individuals at any given time. Defined for each age group, i=1 to 13. |
| c_i,j_ | Contact rate between age groups i (i=01 to 13) and age group j (j=01 to 13) | Polish POLYMOD matrix [1] adjusted for average state-specific household size. See Methods and Technical Appendix, Section #1. |
| pi | Transmission probability per contact | Initial values for each state based on [3] and then adjusted during model fitting, see Technical Appendix, Section #1. Defined for each age group, i=1 to 13. |
| g | 1/infectious period | 1/25 days (21-28). Initial values based on [23] and then adjusted during model fitting. See Technical Appendix, Section #1 and #6. Assumed as the same for primary and secondary infections when applicable, i.e. models SIRS/SIRSIs. |
| wi | Waning of infection induced immunity | 25 years (10-50). Derived during model fitting, see Technical Appendix, Section #1 and #6. |
| mp | Pertussis-specific mortality | Mortality rate from pertussis, state-specific population data from the National Institute of Geography and Statistics and pertussis-specific mortality data from National Mortality System. See Technical Appendix, Section #2. |
| r | Reporting probability | State-specific likelihood that an infection will be symptomatic/reported. Derived during model fitting, see Technical Appendix, Section #1 and #6. The reporting rate for secondary infections, applicable to model structure SIRSI, was assumed to be 1/10 of that of the primary infection. |
| Vaccine | |  |
| Vaccine coverage | |  |
| vc1i | Vaccine coverage wP, 1^st^ dose | Probability of being vaccinated, estimated from the National Survey of Vaccine Coverage in State Capitals in Brazil in 2007. Estimated for each applicable age group i. See Technical Appendix for details, Section #3. |
| vc2i | Vaccine coverage wP, 2^nd^ dose | Probability of being vaccinated, estimated from the National Survey of Vaccine Coverage in State Capitals in Brazil in 2007. Estimated for each applicable age group i.See Technical Appendix for details, Section #3. |
| vc3i | Vaccine coverage wP, 3^rd^ dose | Probability of being vaccinated, estimated from the National Survey of Vaccine Coverage in State Capitals in Brazil in 2007. Estimated for each applicable age group i.See Technical Appendix for details, Section #3. |
| vcm | Vaccine coverage aP, maternal | State-specific data from the Brazilian National Immunization Program, Section #3. |
| Vaccine effectiveness | |  |
| ve1 | Effectiveness wP, 1^st^ dose | 0.68 (95%CI: 0.456 - 0.811) [14, 24] |
| ve2 | Effectiveness wP, 2^nd^ dose | 0.92 (95% CI: 0.847 - 0.957) [14, 24] |
| ve3 | Effectiveness wP, 3^rd^ dose | 0.99 (95% CI: 0.989 - 1.000) [14, 24] |
| vem | Effectiveness aP, maternal | 0.91 (95% CI:0.82 - 0.95) [25] |
| Primary vaccine failure | |  |
| vfw | Proportion of vaccine failure, wP | 0.1 (0 - 0.15) [26] |
| vfa | Proportion of vaccine failure, aP | 0.1 (0 - 0.15) [2] |
| Immunity waning | |  |
| w1 | Waning immunity of wP 1^st^ dose | 14.5 years (range 5-30 years). Derived during model fitting, see Technical Appendix, Section #1 and #6. |
| w2 | Waning immunity of wP 2^nd^ dose | 14.5 years (range 5-30 years). Derived during model fitting, see Technical Appendix, Section #1 and #6. |
| w3 | Waning immunity of wP 3^rd^ dose | 14.5 years (range 5-30 years). Derived during model fitting, see Technical Appendix, Section #1 and #6. |
| wm | Waning immunity of aP, infant* | 3 months (range 2-4 months) [2] |

**9. Model equations by age group**

**Age group #01 [0-1) months: Sp01, Ip01, R01, Ss01, Is01, Vmp01, Vmn01**

- **Births occur to Sp compartment as well as Vmp and Vmn when maternal vaccine is applied**
- **Vaccine protection (vp) is defined by effectiveness * (1 – failure): vpm=vem*(1-vfa)**

$$f01=c_{01,01}*p1*\frac{\left( Ip01+Is01 \right)}{N01}+c_{01,02}*p2*\frac{\left( Ip02+Is02 \right)}{N02}+c_{01,03}*p3*\frac{\left( Ip03+Is03 \right)}{N03}+$$

$$c_{01,04}*p4*\frac{\left( Ip04+Is04 \right)}{N04}+c_{01,05}*p5*\frac{\left( Ip05+Is05 \right)}{N05}+c_{01,06}*p6*\frac{\left( Ip06+Is06 \right)}{N06}+$$

$$c_{01,07}*p7*\frac{\left( Ip07+Is07 \right)}{N07}+c_{01,08}*p8*\frac{\left( Ip08+Is08 \right)}{N08}+c_{01,09}*p9*\frac{\left( Ip09+Is09 \right)}{N09}+$$

$$c_{01,10}*p10*\frac{\left( Ip10+Is10 \right)}{N10}+c_{01,11}*p11*\frac{\left( Ip11+Is11 \right)}{N11}+c_{01,12}*p12*\frac{\left( Ip12+Is12 \right)}{N12}+$$

$$c_{01,13}*p13*\frac{\left( Ip13+Is13 \right)}{N13}$$

$$Sp01=Births*\left( 1-vcm \right)-(d01+m01)*Sp01+wm*Vmp01-f01*Sp01$$

$$Ip01=-\left( d01+m01 \right)*Ip01+f01*\left( Sp01+Vmn01 \right)-g*Ip01$$

$$R01=-\left( d01+m01 \right)*R01+g*(Ip01+Is01)-wi*R01$$

$$Ss01=-\left( d01+m01 \right)*Ss01+wi*R01-f01*Ss01$$

$$Is01=-\left( d01+m01 \right)*Is01+f01*Ss01-g*Is01$$

$$Vmn01=Births*vcm*(1-vpm)-(d01+m01)*Vmn01-f01*Vmn01$$

$$Vmp01=Births*vcm*vpm-\left( d01+m01 \right)*Vmp01-wm*Vmp01$$

**Age group #02 [2-3) months: Sp02, Ip02, R02, Ss02, Is02, Vmp02, Vmn02, V1p02, V1n02**

- **Childhood vaccination starts, 1^st^ dose is given moving infants from Sp, Vmp, and Vmn into V1p and V1n**
- **Vaccine protection (vp) is defined by effectiveness * (1 – failure): vp1=ve1*(1-vfw)**

$$f02=c_{02,01}*p1*\frac{\left( Ip01+Is01 \right)}{N01}+c_{02,02}*p2*\frac{\left( Ip02+Is02 \right)}{N02}+c_{02,03}*p3*\frac{\left( Ip03+Is03 \right)}{N03}+$$

$$c_{02,04}*p4*\frac{\left( Ip04+Is04 \right)}{N04}+c_{02,05}*p5*\frac{\left( Ip05+Is05 \right)}{N05}+c_{02,06}*p6*\frac{\left( Ip06+Is06 \right)}{N06}+$$

$$c_{02,07}*p7*\frac{\left( Ip07+Is07 \right)}{N07}+c_{02,08}*p8*\frac{\left( Ip08+Is08 \right)}{N08}+c_{02,09}*p9*\frac{\left( Ip09+Is09 \right)}{N09}+$$

$$c_{02,10}*p10*\frac{\left( Ip10+Is10 \right)}{N10}+c_{02,11}*p11*\frac{\left( Ip11+Is11 \right)}{N11}+c_{02,12}*p12*\frac{\left( Ip12+Is12 \right)}{N12}+$$

$$c_{02,13}*p13*\frac{\left( Ip13+Is13 \right)}{N13}$$

$$Sp02=d01*Sp01-\left( d02+m02 \right)*Sp02+wm*Vmp02+w1*V1p02-f02*Sp02-vc102*Sp02$$

$$Ip02=d01*Ip01-\left( d02+m02 \right)*Ip02+f02*\left( Sp02+Vmn02+V1n02 \right)-g*Ip02$$

$$R02=d01*R01-\left( d02+m02 \right)*R02+g*\left( Ip02+Is02 \right)-wi*R02$$

$$Ss02=d01*Ss01-\left( d02+m02 \right)*Ss02+wi*R02-f02*Ss02$$

$$Is02=d01*Is01-\left( d02+m02 \right)*Is02+f02*Ss02-g*Is02$$

$$Vmn02=d01*Vmn01-(d02+m02)*Vmn02-f02*Vmn02-vc102*Vmn02$$

$$Vmp02=d01*Vmp01-\left( d02+m02 \right)*Vmp02-wm*Vmp02-vc102*Vmp02$$

$$V1n02=-\left( d02+m02 \right)*V1n02+vc102*(1-vp1)*Sp02+vc102*(1-vp1)*Vmn02+vc102*(1-vp1)*Vmp02-f02*V1n02$$

$$V1p02=-\left( d02+m02 \right)*V1p02+vc102*vp1*Sp02+vc102*vp1*Vmn02+vc102*vp1*Vmp02-w1*V1p02$$

**Age group #03 [4-5) months: Sp03, Ip03, R03, Ss03, Is03, Vmp03, Vmn03, V1p03, V1n03, V2p03, V2n03**

- **Childhood vaccination continues, 1^st^ dose is given moving infants from Sp, Vmp, and Vmn into V1p and V1n, 2^nd^ dose is given moving infants from V1p and V1n into V2p and V2n**
- **Vaccine protection (vp)=effectiveness * (1 – failure): vp1=ve1*(1-vfw); vp2=ve2*(1-vfw)**

$$f03=c_{03,01}*p1*\frac{\left( Ip01+Is01 \right)}{N01}+c_{03,02}*p2*\frac{\left( Ip02+Is02 \right)}{N02}+c_{03,03}*p3*\frac{\left( Ip03+Is03 \right)}{N03}+$$

$$c_{03,04}*p4*\frac{\left( Ip04+Is04 \right)}{N04}+c_{03,05}*p5*\frac{\left( Ip05+Is05 \right)}{N05}+c_{03,06}*p6*\frac{\left( Ip06+Is06 \right)}{N06}+$$

$$c_{03,07}*p7*\frac{\left( Ip07+Is07 \right)}{N07}+c_{03,08}*p8*\frac{\left( Ip08+Is08 \right)}{N08}+c_{03,09}*p9*\frac{\left( Ip09+Is09 \right)}{N09}+$$

$$c_{03,10}*p10*\frac{\left( Ip10+Is10 \right)}{N10}+c_{03,11}*p11*\frac{\left( Ip11+Is11 \right)}{N11}+c_{03,12}*p12*\frac{\left( Ip12+Is12 \right)}{N12}+$$

$$c_{03,13}*p13*\frac{\left( Ip13+Is13 \right)}{N13}$$

$$Sp03=d02*Sp02-\left( d03+m03 \right)*Sp03+wm*Vmp03+w1*V1p03+w2*V2p03-f03*Sp03-vc103*Sp03$$

$$Ip03=d02*Ip02-\left( d03+m03 \right)*Ip03+f03*\left( Sp03+Vmn03+V1n03+V2n03 \right)-g*Ip03$$

$$R03=d02*R02-\left( d03+m03 \right)*R03+g*\left( Ip03+Is03 \right)-wi*R03$$

$$Ss03=d02*Ss02-\left( d03+m03 \right)*Ss03+wi*R03-f03*Ss03$$

$$Is03=d02*Is02-\left( d03+m03 \right)*Is03+f03*Ss03-g*Is03$$

$$Vmn03=d02*Vmn02-(d03+m03)*Vmn03-f03*Vmn03-vc103*Vmn03$$

$$Vmp03=d02*Vmp02-\left( d03+m03 \right)*Vmp03-wm*Vmp03-vc103*Vmp03$$

$$V1n03=d02*V1n02-\left( d03+m03 \right)*V1n03+vc103*(1-vp1)*Sp03+vc103*(1-vp1)*Vmn03+vc103*(1-vp1)*Vmp03-vc203*V1n03-f03*V1n03$$

$$V1p03=d02*V1p02-\left( d03+m03 \right)*V1p03+vc103*vp1*Sp03+vc103*vp1*Vmn03+vc103*vp1*Vmp03-vc203*vp2*V1p03-w1*V1p03$$

$$V2n03=-\left( d03+m03 \right)*V2n03+vc203*(1-vp2)*V1n03-f03*V2n03$$

$$V2p03=-\left( d03+m03 \right)*V2p03+vc203*vp2*V1n03+vc203*vp2*V1p03-w2*V2p03$$

**Age group #04 [6-8) months: Sp04, Ip04, R04, Ss04, Is04, Vmp04, Vmn04, V1p04, V1n04, V2p04, V2n04, V3p04, V3n04**

- **Childhood vaccination continues, 1^st^ dose is given moving infants from Sp, Vmp, and Vmn into V1p and V1n, 2^nd^ dose is given moving infants from V1p and V1n into V2p and V2n, 3^rd^ dose is given moving infants from V2p and V2n into V3p and V3n**
- **Vaccine protection (vp): vp1=ve1*(1-vfw); vp2=ve2*(1-vfw); vp3=ve3*(1-vfw)**

$$f04=c_{04,01}*p1*\frac{\left( Ip01+Is01 \right)}{N01}+c_{04,02}*p2*\frac{\left( Ip02+Is02 \right)}{N02}+c_{04,03}*p3*\frac{\left( Ip03+Is03 \right)}{N03}+$$

$$c_{04,04}*p4*\frac{\left( Ip04+Is04 \right)}{N04}+c_{04,05}*p5*\frac{\left( Ip05+Is05 \right)}{N05}+c_{04,06}*p6*\frac{\left( Ip06+Is06 \right)}{N06}+$$

$$c_{04,07}*p7*\frac{\left( Ip07+Is07 \right)}{N07}+c_{04,08}*p8*\frac{\left( Ip08+Is08 \right)}{N08}+c_{04,09}*p9*\frac{\left( Ip09+Is09 \right)}{N09}+$$

$$c_{04,10}*p10*\frac{\left( Ip10+Is10 \right)}{N10}+c_{04,11}*p11*\frac{\left( Ip11+Is11 \right)}{N11}+c_{04,12}*p12*\frac{\left( Ip12+Is12 \right)}{N12}+$$

$$c_{04,13}*p13*\frac{\left( Ip13+Is13 \right)}{N13}$$

$$Sp04=d03*Sp03-\left( d04+m04 \right)*Sp04+wm*Vmp04+w1*V1p04+w2*V2p04+w3*V3p04-f04*Sp04-vc104*Sp04$$

$$Ip04=d03*Ip03-\left( d04+m04 \right)*Ip04+f04*\left( Sp04+Vmn04+V1n04+V2n04+V3n04 \right)-g*Ip04$$

$$R04=d03*R03-\left( d04+m04 \right)*R04+g*\left( Ip04+Is04 \right)-wi*R04$$

$$Ss04=d03*Ss03-\left( d04+m04 \right)*Ss04+wi*R04-f04*Ss04$$

$$Is04=d03*Is03-\left( d04+m04 \right)*Is04+f04*Ss04-g*Is04$$

$$Vmn04=d03*Vmn03-(d04+m04)*Vmn04-f04*Vmn04-vc104*Vmn04$$

$$Vmp04=d03*Vmp03-\left( d04+m04 \right)*Vmp04-wm*Vmp04-vc104*Vmp04$$

$$V1n04=d03*V1n03-\left( d04+m04 \right)*V1n04+vc104*(1-vp1)*Sp04+vc104*(1-vp1)*Vmn04+vc104*(1-vp1)*Vmp04-vc204*V1n04-f04*V1n04$$

$$V1p04=d03*V1p03-\left( d04+m04 \right)*V1p04+vc104*vp1*Sp04+vc104*vp1*Vmn04+vc104*vp1*Vmp04-vc204*vp2*V1p04-w1*V1p04$$

$$V2n04=d03*V2n03-\left( d04+m04 \right)*V2n04+vc204*\left( 1-vp2 \right)*V1n04-vc304*V2n04-f04*V2n04$$

$$V2p04=d03*V2p03-\left( d04+m04 \right)*V2p04+vc204*vp2*V1n04+vc204*vp2*V1p04-vc304*vp3*V2p04-w2*V2p04$$

$$V3n04=-\left( d04+m04 \right)*V3n04+vc304*(1-vp3)*V2n04-f04*V3n04$$

$$V3p04=-\left( d04+m04 \right)*V3p04+vc304*vp3*V2n04+vc304*vp3*V2p04-w3*V3p04$$

**Age group #05 [9-11) months: Sp05, Ip05, R05, Ss05, Is05, Vmp05, Vmn05, V1p05, V1n05, V2p05, V2n05, V3p05, V3n05**

- **Childhood vaccination continues, 1^st^ dose is given moving infants from Sp, Vmp, and Vmn into V1p and V1n, 2^nd^ dose is given moving infants from V1p and V1n into V2p and V2n, 3^rd^ dose is given moving infants from V2p and V2n into V3p and V3n**
- **Vaccine protection (vp): vp1=ve1*(1-vfw); vp2=ve2*(1-vfw); vp3=ve3*(1-vfw)**

$$f05=c_{05,01}*p1*\frac{\left( Ip01+Is01 \right)}{N01}+c_{05,02}*p2*\frac{\left( Ip02+Is02 \right)}{N02}+c_{05,03}*p3*\frac{\left( Ip03+Is03 \right)}{N03}+$$

$$c_{05,04}*p4*\frac{\left( Ip04+Is04 \right)}{N04}+c_{05,05}*p5*\frac{\left( Ip05+Is05 \right)}{N05}+c_{05,06}*p6*\frac{\left( Ip06+Is06 \right)}{N06}+$$

$$c_{05,07}*p7*\frac{\left( Ip07+Is07 \right)}{N07}+c_{05,08}*p8*\frac{\left( Ip08+Is08 \right)}{N08}+c_{05,09}*p9*\frac{\left( Ip09+Is09 \right)}{N09}+$$

$$c_{05,10}*p10*\frac{\left( Ip10+Is10 \right)}{N10}+c_{05,11}*p11*\frac{\left( Ip11+Is11 \right)}{N11}+c_{05,12}*p12*\frac{\left( Ip12+Is12 \right)}{N12}+$$

$$c_{05,13}*p13*\frac{\left( Ip13+Is13 \right)}{N13}$$

$$Sp05=d04*Sp04-\left( d05+m05 \right)*Sp05+wm*Vmp05+w1*V1p05+w2*V2p05+w3*V3p05-f05*Sp05-vc105*Sp05$$

$$Ip05=d04*Ip04-\left( d05+m05 \right)*Ip05+f05*\left( Sp05+Vmn05+V1n05+V2n05+V3n05 \right)-g*Ip05$$

$$R05=d04*R04-\left( d05+m05 \right)*R05+g*\left( Ip05+Is05 \right)-wi*R05$$

$$Ss05=d04*Ss04-\left( d05+m05 \right)*Ss05+wi*R05-f05*Ss05$$

$$Is05=d04*Is04-\left( d05+m05 \right)*Is05+f05*Ss05-g*Is05$$

$$Vmn05=d04*Vmn04-(d05+m05)*Vmn05-f05*Vmn05-vc105*Vmn05$$

$$Vmp05=d04*Vmp04-\left( d05+m05 \right)*Vmp05-wm*Vmp05-vc105*Vmp05$$

$$V1n05=d04*V1n04-\left( d05+m05 \right)*V1n05+vc105*(1-vp1)*Sp05+vc105*(1-vp1)*Vmn05+vc105*(1-vp1)*Vmp05-vc205*V1n05-f05*V1n05$$

$$V1p05=d04*V1p04-\left( d05+m05 \right)*V1p05+vc105*vp1*Sp05+vc105*vp1*Vmn05+vc105*vp1*Vmp05-vc205*vp2*V1p05-w1*V1p05$$

$$V2n05=d04*V2n04-\left( d05+m05 \right)*V2n05+vc205*\left( 1-vp2 \right)*V1n05-vc305*V2n05-f05*V2n05$$

$$V2p05=d04*V2p04-\left( d05+m05 \right)*V2p05+vc205*vp2*V1n05+vc205*vp2*V1p05-vc305*vp3*V2p05-w2*V2p05$$

$$V3n05=d04*V3n04-\left( d05+m05 \right)*V3n05+vc305*(1-vp3)*V2n05-f05*V3n05$$

$$V3p05=d04*V3p04-\left( d05+m05 \right)*V3p05+vc305*vp3*V2n05+vc305*vp3*V2p05-w3*V3p05$$

**Age group #06 [12-23) months: Sp06, Ip06, R06, Ss06, Is06, Vmp06, Vmn06, V1p06, V1n06, V2p06, V2n06, V3p06, V3n06**

- **Childhood vaccination continues, 1^st^ dose is given moving infants from Sp, Vmp, and Vmn into V1p and V1n, 2^nd^ dose is given moving infants from V1p and V1n into V2p and V2n, 3^rd^ dose is given moving infants from V2p and V2n into V3p and V3n**
- **Vaccine protection (vp): vp1=ve1*(1-vfw); vp2=ve2*(1-vfw); vp3=ve3*(1-vfw)**

$$f06=c_{06,01}*p1*\frac{\left( Ip01+Is01 \right)}{N01}+c_{06,02}*p2*\frac{\left( Ip02+Is02 \right)}{N02}+c_{06,03}*p3*\frac{\left( Ip03+Is03 \right)}{N03}+$$

$$c_{06,04}*p4*\frac{\left( Ip04+Is04 \right)}{N04}+c_{06,05}*p5*\frac{\left( Ip05+Is05 \right)}{N05}+c_{06,06}*p6*\frac{\left( Ip06+Is06 \right)}{N06}+$$

$$c_{06,07}*p7*\frac{\left( Ip07+Is07 \right)}{N07}+c_{06,08}*p8*\frac{\left( Ip08+Is08 \right)}{N08}+c_{06,09}*p9*\frac{\left( Ip09+Is09 \right)}{N09}+$$

$$c_{06,10}*p10*\frac{\left( Ip10+Is10 \right)}{N10}+c_{06,11}*p11*\frac{\left( Ip11+Is11 \right)}{N11}+c_{06,12}*p12*\frac{\left( Ip12+Is12 \right)}{N12}+$$

$$c_{06,13}*p13*\frac{\left( Ip13+Is13 \right)}{N13}$$

$$Sp06=d05*Sp05-\left( d06+m06 \right)*Sp06+wm*Vmp06+w1*V1p06+w2*V2p06+w3*V3p06-f06*Sp06-vc106*Sp06$$

$$Ip06=d05*Ip05-\left( d06+m06 \right)*Ip06+f06*\left( Sp06+Vmn06+V1n06+V2n06+V3n06 \right)-g*Ip06$$

$$R06=d05*R05-\left( d06+m06 \right)*R06+g*(Ip06+Is06)-wi*R06$$

$$Ss06=d05*Ss05-\left( d06+m06 \right)*Ss06+wi*R06-f06*Ss06$$

$$Is06=d05*Is05-\left( d06+m06 \right)*Is06+f06*Ss06-g*Is06$$

$$Vmn06=d05*Vmn05-(d06+m06)*Vmn06-f06*Vmn06-vc106*Vmn06$$

$$Vmp06=d05*Vmp05-\left( d06+m06 \right)*Vmp06-wm*Vmp06-vc106*Vmp06$$

$$V1n06=d05*V1n05-\left( d06+m06 \right)*V1n06+vc106*(1-vp1)*Sp06+vc106*(1-vp1)*Vmn06+vc106*(1-vp1)*Vmp06-vc206*V1n06-f06*V1n06$$

$$V1p06=d05*V1p05-\left( d06+m06 \right)*V1p06+vc106*vp1*Sp06+vc106*vp1*Vmn06+vc106*vp1*Vmp06-vc206*vp2*V1p06-w1*V1p06$$

$$V2n06=d05*V2n05-\left( d06+m06 \right)*V2n06+vc206*\left( 1-vp2 \right)*V1n06-vc306*V2n06-f06*V2n06$$

$$V2p06=d05*V2p05-\left( d06+m06 \right)*V2p06+vc206*vp2*V1n06+vc206*vp2*V1p06-vc306*vp3*V2p06-w2*V2p06$$

$$V3n06=d05*V3n05-\left( d06+m06 \right)*V3n06+vc306*(1-vp3)*V2n06-f06*V3n06$$

$$V3p06=d05*V3p05-\left( d06+m06 \right)*V3p06+vc306*vp3*V2n06+vc306*vp3*V2p06-w3*V3p06$$

**Age group #07 [2-4) years: Sp07, Ip07, R07, Ss07, Is07, Vmp07, Vmn07, V1p07, V1n07, V2p07, V2n07, V3p07, V3n07**

- **Vaccines no longer applied.**

$$f07=c_{07,01}*p1*\frac{\left( Ip01+Is01 \right)}{N01}+c_{07,02}*p2*\frac{\left( Ip02+Is02 \right)}{N02}+c_{07,03}*p3*\frac{\left( Ip03+Is03 \right)}{N03}+$$

$$c_{07,04}*p4*\frac{\left( Ip04+Is04 \right)}{N04}+c_{07,05}*p5*\frac{\left( Ip05+Is05 \right)}{N05}+c_{07,06}*p6*\frac{\left( Ip06+Is06 \right)}{N06}+$$

$$c_{07,07}*p7*\frac{\left( Ip07+Is07 \right)}{N07}+c_{07,08}*p8*\frac{\left( Ip08+Is08 \right)}{N08}+c_{07,09}*p9*\frac{\left( Ip09+Is09 \right)}{N09}+$$

$$c_{07,10}*p10*\frac{\left( Ip10+Is10 \right)}{N10}+c_{07,11}*p11*\frac{\left( Ip11+Is11 \right)}{N11}+c_{07,12}*p12*\frac{\left( Ip12+Is12 \right)}{N12}+$$

$$c_{07,13}*p13*\frac{\left( Ip13+Is13 \right)}{N13}$$

$$Sp07=d06*Sp06-\left( d07+m07 \right)*Sp07+wm*Vmp07+w1*V1p07+w2*V2p07+w3*V3p07-f07*Sp07$$

$$Ip07=d06*Ip06-\left( d07+m07 \right)*Ip07+f07*\left( Sp07+Vmn07+V1n07+V2n07+V3n07 \right)-g*Ip07$$

$$R07=d06*R06-\left( d07+m07 \right)*R07+g*(Ip07+Is07)-wi*R07$$

$$Ss07=d06*Ss06-\left( d07+m07 \right)*Ss07+wi*R07-f07*Ss07$$

$$Is07=d06*Is06-\left( d07+m07 \right)*Is07+f07*Ss07-g*Is07$$

$$Vmn07=d06*Vmn06-(d07+m07)*Vmn07-f07*Vmn07$$

$$Vmp07=d06*Vmp06-\left( d07+m07 \right)*Vmp07-wm*Vmp07$$

$$V1n07=d06*V1n06-\left( d07+m07 \right)*V1n07-f07*V1n07$$

$$V1p07=d06*V1p06-\left( d07+m07 \right)*V1p07-w1*V1p07$$

$$V2n07=d06*V2n06-\left( d07+m07 \right)*V2n07-f07*V2n07$$

$$V2p07=d06*V2p06-\left( d07+m07 \right)*V2p07-w2*V2p07$$

$$V3n07=d06*V3n06-\left( d07+m07 \right)*V3n07-f07*V3n07$$

$$V3p07=d06*V3p06-\left( d07+m07 \right)*V3p07-w3*V3p07$$

**Age group #08 [5-9) years: Sp08, Ip08, R08, Ss08, Is08, Vmp08, Vmn08, V1p08, V1n08, V2p08, V2n08, V3p08, V3n08**

- **Vaccines no longer applied.**

$$f08=c_{08,01}*p1*\frac{\left( Ip01+Is01 \right)}{N01}+c_{08,02}*p2*\frac{\left( Ip02+Is02 \right)}{N02}+c_{08,03}*p3*\frac{\left( Ip03+Is03 \right)}{N03}+$$

$$c_{08,04}*p4*\frac{\left( Ip04+Is04 \right)}{N04}+c_{08,05}*p5*\frac{\left( Ip05+Is05 \right)}{N05}+c_{08,06}*p6*\frac{\left( Ip06+Is06 \right)}{N06}+$$

$$c_{08,07}*p7*\frac{\left( Ip07+Is07 \right)}{N07}+c_{08,08}*p8*\frac{\left( Ip08+Is08 \right)}{N08}+c_{08,09}*p9*\frac{\left( Ip09+Is09 \right)}{N09}+$$

$$c_{08,10}*p10*\frac{\left( Ip10+Is10 \right)}{N10}+c_{08,11}*p11*\frac{\left( Ip11+Is11 \right)}{N11}+c_{08,12}*p12*\frac{\left( Ip12+Is12 \right)}{N12}+$$

$$c_{08,13}*p13*\frac{\left( Ip13+Is13 \right)}{N13}$$

$$Sp08=d07*Sp07-\left( d08+m08 \right)*Sp08+wm*Vmp08+w1*V1p08+w2*V2p08+w3*V3p08-f08*Sp08$$

$$Ip08=d07*Ip07-\left( d08+m08 \right)*Ip08+f08*\left( Sp08+Vmn08+V1n08+V2n08+V3n08 \right)-g*Ip08$$

$$R08=d07*R07-\left( d08+m08 \right)*R08+g*(Ip08+Is08)-wi*R08$$

$$Ss08=d07*Ss07-\left( d08+m08 \right)*Ss08+wi*R08-f08*Ss08$$

$$Is08=d07*Is07-\left( d08+m08 \right)*Is08+f08*Ss08-g*Is08$$

$$Vmn08=d07*Vmn07-(d08+m08)*Vmn08-f08*Vmn08$$

$$Vmp08=d07*Vmp07-\left( d08+m08 \right)*Vmp08-wm*Vmp08$$

$$V1n08=d07*V1n07-\left( d08+m08 \right)*V1n08-f08*V1n08$$

$$V1p08=d07*V1p07-\left( d08+m08 \right)*V1p08-w1*V1p08$$

$$V2n08=d07*V2n07-\left( d08+m08 \right)*V2n08-f08*V2n08$$

$$V2p08=d07*V2p07-\left( d08+m08 \right)*V2p08-w2*V2p08$$

$$V3n08=d07*V3n07-\left( d08+m08 \right)*V3n08-f08*V3n08$$

$$V3p08=d07*V3p07-\left( d08+m08 \right)*V3p08-w3*V3p08$$

**Age group #09 [10-14) years: Sp09, Ip09, R09, Ss09, Is09, Vmp09, Vmn09, V1p09, V1n09, V2p09, V2n09, V3p09, V3n09**

- **Vaccines no longer applied.**

$$f09=c_{09,01}*p1*\frac{\left( Ip01+Is01 \right)}{N01}+c_{09,02}*p2*\frac{\left( Ip02+Is02 \right)}{N02}+c_{09,03}*p3*\frac{\left( Ip03+Is03 \right)}{N03}+$$

$$c_{09,04}*p4*\frac{\left( Ip04+Is04 \right)}{N04}+c_{09,05}*p5*\frac{\left( Ip05+Is05 \right)}{N05}+c_{09,06}*p6*\frac{\left( Ip06+Is06 \right)}{N06}+$$

$$c_{09,07}*p7*\frac{\left( Ip07+Is07 \right)}{N07}+c_{09,08}*p8*\frac{\left( Ip08+Is08 \right)}{N08}+c_{09,09}*p9*\frac{\left( Ip09+Is09 \right)}{N09}+$$

$$c_{09,10}*p10*\frac{\left( Ip10+Is10 \right)}{N10}+c_{09,11}*p11*\frac{\left( Ip11+Is11 \right)}{N11}+c_{09,12}*p12*\frac{\left( Ip12+Is12 \right)}{N12}+$$

$$c_{09,13}*p13*\frac{\left( Ip13+Is13 \right)}{N13}$$

$$Sp09=d08*Sp08-\left( d09+m09 \right)*Sp09+wm*Vmp09+w1*V1p09+w2*V2p09+w3*V3p09-f09*Sp09$$

$$Ip09=d08*Ip08-\left( d09+m09 \right)*Ip09+f09*\left( Sp09+Vmn09+V1n09+V2n09+V3n09 \right)-g*Ip09$$

$$R09=d08*R08-\left( d09+m09 \right)*R09+g*(Ip09+Is09)-wi*R09$$

$$Ss09=d08*Ss08-\left( d09+m09 \right)*Ss09+wi*R09-f09*Ss09$$

$$Is09=d08*Is08-\left( d09+m09 \right)*Is09+f09*Ss09-g*Is09$$

$$Vmn09=d08*Vmn08-(d09+m09)*Vmn09-f09*Vmn09$$

$$Vmp09=d08*Vmp08-\left( d09+m09 \right)*Vmp09-wm*Vmp09$$

$$V1n09=d08*V1n08-\left( d09+m09 \right)*V1n09-f09*V1n09$$

$$V1p09=d08*V1p08-\left( d09+m09 \right)*V1p09-w1*V1p09$$

$$V2n09=d08*V2n08-\left( d09+m09 \right)*V2n09-f09*V2n09$$

$$V2p09=d08*V2p08-\left( d09+m09 \right)*V2p09-w2*V2p09$$

$$V3n09=d08*V3n08-\left( d09+m09 \right)*V3n09-f09*V3n09$$

$$V3p09=d08*V3p08-\left( d09+m09 \right)*V3p09-w3*V3p09$$

**Age group #10 [15-19) years: Sp10, Ip10, R10, Ss10, Is10, Vmp10, Vmn10, V1p10, V1n10, V2p10, V2n10, V3p10, V3n10**

- **Vaccines no longer applied.**

$$f10=c_{10,01}*p1*\frac{\left( Ip01+Is01 \right)}{N01}+c_{10,02}*p2*\frac{\left( Ip02+Is02 \right)}{N02}+c_{10,03}*p3*\frac{\left( Ip03+Is03 \right)}{N03}+$$

$$c_{10,04}*p4*\frac{\left( Ip04+Is04 \right)}{N04}+c_{10,05}*p5*\frac{\left( Ip05+Is05 \right)}{N05}+c_{10,06}*p6*\frac{\left( Ip06+Is06 \right)}{N06}+$$

$$c_{10,07}*p7*\frac{\left( Ip07+Is07 \right)}{N07}+c_{10,08}*p8*\frac{\left( Ip08+Is08 \right)}{N08}+c_{10,09}*p9*\frac{\left( Ip09+Is09 \right)}{N09}+$$

$$c_{10,10}*p10*\frac{\left( Ip10+Is10 \right)}{N10}+c_{10,11}*p11*\frac{\left( Ip11+Is11 \right)}{N11}+c_{10,12}*p12*\frac{\left( Ip12+Is12 \right)}{N12}+$$

$$c_{10,13}*p13*\frac{\left( Ip13+Is13 \right)}{N13}$$

$$Sp10=d09*Sp09-\left( d10+m10 \right)*Sp10+wm*Vmp10+w1*V1p10+w2*V2p10+w3*V3p10-f10*Sp10$$

$$Ip10=d09*Ip09-\left( d10+m10 \right)*Ip10+f10*\left( Sp10+Vmn10+V1n10+V2n10+V3n10 \right)-g*Ip10$$

$$R10=d09*R09-\left( d10+m10 \right)*R10+g*(Ip10+Is10)-wi*R10$$

$$Ss10=d09*Ss09-\left( d10+m10 \right)*Ss10+wi*R10-f10*Ss10$$

$$Is10=d09*Is09-\left( d10+m10 \right)*Is10+f10*Ss10-g*Is10$$

$$Vmn10=d09*Vmn09-(d10+m10)*Vmn10-f10*Vmn10$$

$$Vmp10=d09*Vmp09-\left( d10+m10 \right)*Vmp10-wm*Vmp10$$

$$V1n10=d09*V1n09-\left( d10+m10 \right)*V1n10-f10*V1n10$$

$$V1p10=d09*V1p09-\left( d10+m10 \right)*V1p10-w1*V1p10$$

$$V2n10=d09*V2n09-\left( d10+m10 \right)*V2n10-f10*V2n10$$

$$V2p10=d09*V2p09-\left( d10+m10 \right)*V2p10-w2*V2p10$$

$$V3n10=d09*V3n09-\left( d10+m10 \right)*V3n10-f10*V3n10$$

$$V3p10=d09*V3p09-\left( d10+m10 \right)*V3p10-w3*V3p10$$

**Age group #11 [20-49) years: Sp11, Ip11, R11, Ss11, Is11, Vmp11, Vmn11, V1p11, V1n11, V2p11, V2n11, V3p11, V3n11**

- **Vaccines no longer applied.**

$$f11=c_{11,01}*p1*\frac{\left( Ip01+Is01 \right)}{N01}+c_{11,02}*p2*\frac{\left( Ip02+Is02 \right)}{N02}+c_{11,03}*p3*\frac{\left( Ip03+Is03 \right)}{N03}+$$

$$c_{11,04}*p4*\frac{\left( Ip04+Is04 \right)}{N04}+c_{11,05}*p5*\frac{\left( Ip05+Is05 \right)}{N05}+c_{11,06}*p6*\frac{\left( Ip06+Is06 \right)}{N06}+$$

$$c_{11,07}*p7*\frac{\left( Ip07+Is07 \right)}{N07}+c_{11,08}*p8*\frac{\left( Ip08+Is08 \right)}{N08}+c_{11,09}*p9*\frac{\left( Ip09+Is09 \right)}{N09}+$$

$$c_{11,10}*p10*\frac{\left( Ip10+Is10 \right)}{N10}+c_{11,11}*p11*\frac{\left( Ip11+Is11 \right)}{N11}+c_{11,12}*p12*\frac{\left( Ip12+Is12 \right)}{N12}+$$

$$c_{11,13}*p13*\frac{\left( Ip13+Is13 \right)}{N13}$$

$$Sp11=d10*Sp10-\left( d11+m11 \right)*Sp11+wm*Vmp11+w1*V1p11+w2*V2p11+w3*V3p11-f11*Sp11$$

$$Ip11=d10*Ip10-\left( d11+m11 \right)*Ip11+f11*\left( Sp11+Vmn11+V1n11+V2n11+V3n11 \right)-g*Ip11$$

$$R11=d10*R10-\left( d11+m11 \right)*R11+g*(Ip11+Is11)-wi*R11$$

$$Ss11=d10*Ss10-\left( d11+m11 \right)*Ss11+wi*R11-f11*Ss11$$

$$Is11=d10*Is10-\left( d11+m11 \right)*Is11+f11*Ss11-g*Is11$$

$$Vmn11=d10*Vmn10-(d11+m11)*Vmn11-f11*Vmn11$$

$$Vmp11=d10*Vmp10-\left( d11+m11 \right)*Vmp11-wm*Vmp11$$

$$V1n11=d10*V1n10-\left( d11+m11 \right)*V1n11-f11*V1n11$$

$$V1p11=d10*V1p10-\left( d11+m11 \right)*V1p11-w1*V1p11$$

$$V2n11=d10*V2n10-\left( d11+m11 \right)*V2n11-f11*V2n11$$

$$V2p11=d10*V2p10-\left( d11+m11 \right)*V2p11-w2*V2p11$$

$$V3n11=d10*V3n10-\left( d11+m11 \right)*V3n11-f11*V3n11$$

$$V3p11=d10*V3p10-\left( d11+m11 \right)*V3p11-w3*V3p11$$

**Age group #12 [50-79) years: Sp12, Ip12, R12, Ss12, Is12, Vmp12, Vmn12, V1p12, V1n12, V2p12, V2n12, V3p12, V3n12**

- **Vaccines no longer applied.**

$$f12=c_{12,01}*p1*\frac{\left( Ip01+Is01 \right)}{N01}+c_{12,02}*p2*\frac{\left( Ip02+Is02 \right)}{N02}+c_{12,03}*p3*\frac{\left( Ip03+Is03 \right)}{N03}+$$

$$c_{12,04}*p4*\frac{\left( Ip04+Is04 \right)}{N04}+c_{12,05}*p5*\frac{\left( Ip05+Is05 \right)}{N05}+c_{12,06}*p6*\frac{\left( Ip06+Is06 \right)}{N06}+$$

$$c_{12,07}*p7*\frac{\left( Ip07+Is07 \right)}{N07}+c_{12,08}*p8*\frac{\left( Ip08+Is08 \right)}{N08}+c_{12,09}*p9*\frac{\left( Ip09+Is09 \right)}{N09}+$$

$$c_{12,10}*p10*\frac{\left( Ip10+Is10 \right)}{N10}+c_{12,11}*p11*\frac{\left( Ip11+Is11 \right)}{N11}+c_{12,12}*p12*\frac{\left( Ip12+Is12 \right)}{N12}+$$

$$c_{12,13}*p13*\frac{\left( Ip13+Is13 \right)}{N13}$$

$$Sp12=d11*Sp11-\left( d12+m12 \right)*Sp12+wm*Vmp12+w1*V1p12+w2*V2p12+w3*V3p12-f12*Sp12$$

$$Ip12=d11*Ip11-\left( d12+m12 \right)*Ip12+f12*\left( Sp12+Vmn12+V1n12+V2n12+V3n12 \right)-g*Ip12$$

$$R12=d11*R11-\left( d12+m12 \right)*R12+g*(Ip12+Is12)-wi*R12$$

$$Ss12=d11*Ss11-\left( d12+m12 \right)*Ss12+wi*R12-f12*Ss12$$

$$Is12=d11*Is11-\left( d12+m12 \right)*Is12+f12*Ss12-g*Is12$$

$$Vmn12=d11*Vmn11-(d12+m12)*Vmn12-f12*Vmn12$$

$$Vmp12=d11*Vmp11-\left( d12+m12 \right)*Vmp12-wm*Vmp12$$

$$V1n12=d11*V1n11-\left( d12+m12 \right)*V1n12-f12*V1n12$$

$$V1p12=d11*V1p11-\left( d12+m12 \right)*V1p12-w1*V1p12$$

$$V2n12=d11*V2n11-\left( d12+m12 \right)*V2n12-f12*V2n12$$

$$V2p12=d11*V2p11-\left( d12+m12 \right)*V2p12-w2*V2p12$$

$$V3n12=d11*V3n11-\left( d12+m12 \right)*V3n12-f12*V3n12$$

$$V3p12=d11*V3p11-\left( d12+m12 \right)*V3p12-w3*V3p12$$

**Age group #13 [80+ years: Sp13, Ip13, R13, Ss13, Is13, Vmp13, Vmn13, V1p13, V1n13, V2p13, V2n13, V3p13, V3n13**

- **Vaccines no longer applied.**

$$f13=c_{13,01}*p1*\frac{\left( Ip01+Is01 \right)}{N01}+c_{13,02}*p2*\frac{\left( Ip02+Is02 \right)}{N02}+c_{13,03}*p3*\frac{\left( Ip03+Is03 \right)}{N03}+$$

$$c_{13,04}*p4*\frac{\left( Ip04+Is04 \right)}{N04}+c_{13,05}*p5*\frac{\left( Ip05+Is05 \right)}{N05}+c_{13,06}*p6*\frac{\left( Ip06+Is06 \right)}{N06}+$$

$$c_{13,07}*p7*\frac{\left( Ip07+Is07 \right)}{N07}+c_{13,08}*p8*\frac{\left( Ip08+Is08 \right)}{N08}+c_{13,09}*p9*\frac{\left( Ip09+Is09 \right)}{N09}+$$

$$c_{13,10}*p10*\frac{\left( Ip10+Is10 \right)}{N10}+c_{13,11}*p11*\frac{\left( Ip11+Is11 \right)}{N11}+c_{13,12}*p12*\frac{\left( Ip12+Is12 \right)}{N12}+$$

$$c_{13,13}*p13*\frac{\left( Ip13+Is13 \right)}{N13}$$

$$Sp13=d12*Sp12-\left( m13 \right)*Sp13+wm*Vmp13+w1*V1p13+w2*V2p13+w3*V3p13-f13*Sp13$$

$$Ip13=d12*Ip12-\left( m13 \right)*Ip13+f13*\left( Sp13+Vmn13+V1n13+V2n13+V3n13 \right)-g*Ip13$$

$$R13=d12*R12-\left( m13 \right)*R13+g*(Ip13+Is13)-wi*R13$$

$$Ss13=d12*Ss12-\left( m13 \right)*Ss13+wi*R13-f13*Ss13$$

$$Is13=d12*Is12-\left( m13 \right)*Is13+f13*Ss13-g*Is13$$

$$Vmn13=d12*Vmn12-(m13)*Vmn13-f13*Vmn13$$

$$Vmp13=d12*Vmp12-\left( m13 \right)*Vmp13-wm*Vmp13$$

$$V1n13=d12*V1n12-\left( m13 \right)*V1n13-f13*V1n13$$

$$V1p13=d12*V1p12-\left( m13 \right)*V1p13-w1*V1p13$$

$$V2n13=d12*V2n12-\left( m13 \right)*V2n13-f13*V2n13$$

$$V2p13=d12*V2p12-\left( m13 \right)*V2p13-w2*V2p13$$

$$V3n13=d12*V3n12-\left( m13 \right)*V3n13-f13*V3n13$$

$$V3p13=d12*V3p12-\left( m13 \right)*V3p13-w3*V3p13$$

**10. References**

1. Mossong, J., et al., *Social contacts and mixing patterns relevant to the spread of infectious diseases.* PLoS Med, 2008. **5**(3): p. e74.

2. Bento, A.I. and P. Rohani, *Forecasting Epidemiological Consequences of Maternal Immunization.* Clin Infect Dis, 2016. **63**(suppl 4): p. S205-S212.

3. Choi, Y.H., et al., *Investigating the pertussis resurgence in England and Wales, and options for future control.* BMC Med, 2016. **14**(1): p. 121.

4. Blackwood, J.C., et al., *Deciphering the impacts of vaccination and immunity on pertussis epidemiology in Thailand.* Proc Natl Acad Sci U S A, 2013. **110**(23): p. 9595-600.

5. Wearing, H.J. and P. Rohani, *Estimating the duration of pertussis immunity using epidemiological signatures.* PLoS Pathog, 2009. **5**(10): p. e1000647.

6. Crowcroft, N.S., et al., *Deaths from pertussis are underestimated in England.* Arch Dis Child, 2002. **86**(5): p. 336-8.

7. Crowcroft, N.S., et al., *Under-reporting of pertussis in Ontario: A Canadian Immunization Research Network (CIRN) study using capture-recapture.* PLoS One, 2018. **13**(5): p. e0195984.

8. van der Maas, N.A.T., et al., *Severe underestimation of pertussis related hospitalizations and deaths in the Netherlands: A capture-recapture analysis.* Vaccine, 2017. **35**(33): p. 4162-4166.

9. de Melker, H.E., et al., *The incidence of Bordetella pertussis infections estimated in the population from a combination of serological surveys.* J Infect, 2006. **53**(2): p. 106-13.

10. Falleiros Arlant, L.H., et al., *Pertussis in Latin America: epidemiology and control strategies.* Expert Rev Anti Infect Ther, 2014. **12**(10): p. 1265-75.

11. Guimaraes, L.M., E.L. Carneiro, and F.A. Carvalho-Costa, *Increasing incidence of pertussis in Brazil: a retrospective study using surveillance data.* BMC Infect Dis, 2015. **15**: p. 442.

12. Pimentel, A.M., et al., *Pertussis may be the cause of prolonged cough in adolescents and adults in the interepidemic period.* Braz J Infect Dis, 2015. **19**(1): p. 43-6.

13. DeAngelis, H., et al., *Epidemiological and Economic Effects of Priming With the Whole-Cell Bordetella pertussis Vaccine.* JAMA Pediatr, 2016. **170**(5): p. 459-65.

14. Fulton, T.R., et al., *Protective Effect of Contemporary Pertussis Vaccines: A Systematic Review and Meta-analysis.* Clin Infect Dis, 2016. **62**(9): p. 1100-1110.

15. Leite Ida, C., et al., *Burden of disease in Brazil and its regions, 2008.* Cad Saude Publica, 2015. **31**(7): p. 1551-64.

16. Collaborators, G.B.D.B., *Burden of disease in Brazil, 1990-2016: a systematic subnational analysis for the Global Burden of Disease Study 2016.* Lancet, 2018. **392**(10149): p. 760-775.

17. Murray, C.J., et al., *Disability-adjusted life years (DALYs) for 291 diseases and injuries in 21 regions, 1990-2010: a systematic analysis for the Global Burden of Disease Study 2010.* Lancet, 2012. **380**(9859): p. 2197-223.

18. Fernandes, E.G., et al., *Challenges of interpreting epidemiologic surveillance pertussis data with changing diagnostic and immunization practices: the case of the state of Sao Paulo, Brazil.* BMC Infect Dis, 2018. **18**(1): p. 126.

19. Ramsay, J.O. and B.W. Silverman, *Functional data analysis*. 2nd ed. Springer series in statistics. 2005, New York: Springer. xix, 426 p.

20. Brockhaus, S., et al., *Boosting flexible functional regression models with a high number of functional historical effects.* Statistics and Computing, 2017. **27**(4): p. 913-926.

21. Baptista, P.N., V.S. Magalhaes, and L.C. Rodrigues, *The role of adults in household outbreaks of pertussis.* Int J Infect Dis, 2010. **14**(2): p. e111-4.

22. Torres, R.S., et al., *Resurgence of pertussis at the age of vaccination: clinical, epidemiological, and molecular aspects.* J Pediatr (Rio J), 2015. **91**(4): p. 333-8.

23. Atkins, K.E., et al., *Cost-Effectiveness of Pertussis Vaccination During Pregnancy in the United States.* Am J Epidemiol, 2016. **183**(12): p. 1159-70.

24. Juretzko, P., et al., *Effectiveness of acellular pertussis vaccine assessed by hospital-based active surveillance in Germany.* Clin Infect Dis, 2002. **35**(2): p. 162-7.

25. Amirthalingam, G., et al., *Effectiveness of maternal pertussis vaccination in England: an observational study.* Lancet, 2014. **384**(9953): p. 1521-8.

26. Magpantay, F.M., et al., *Pertussis immunity and epidemiology: mode and duration of vaccine-induced immunity.* Parasitology, 2016. **143**(7): p. 835-49.
