# Supplementary material for: Modeling the cost-effectiveness of maternal acellular pertussis immunization (aP) in different socioeconomic settings: A dynamic transmission model of pertussis in three Brazilian states
Source: Vaccine. 2021 Jan 3;39(1):125–36. doi: 10.1016/j.vaccine.2020.09.008 (PMC7738757; doi:10.1016/j.vaccine.2020.09.008)
Supplement: Supplementary data 2 [file mmc2.docx]

**Data statement**

Most of the data used in this study are provided in the Technical Appendices and/or described in Bagattini AM et al., “The data used to build the models: Pertussis morbidity and mortality burden considering various Brazilian data sources,” which has also been submitted for consideration for publication in the Special Issue on Maternal Immunization. Additional data are available upon request from the corresponding author.
